# Transcriptional Responses of *Leptospira interrogans* to Host Innate Immunity: Significant Changes in Metabolism, Oxygen Tolerance, and Outer Membrane

**DOI:** 10.1371/journal.pntd.0000857

**Published:** 2010-10-26

**Authors:** Feng Xue, Haiyan Dong, Jinyu Wu, Zuowei Wu, Weilin Hu, Aihua Sun, Bryan Troxell, X. Frank Yang, Jie Yan

**Affiliations:** 1 State Key Laboratory for Diagnosis and Treatment of Infectious Diseases, First Affiliated Hospital of Medical College, Zhejiang University, Hangzhou, China; 2 Department of Medical Microbiology and Parasitology, Medical College, Zhejiang University School of Medicine, Hangzhou, China; 3 Zhejiang Provincial Key Laboratory of Medical Genetics, Institute of Biomedical Informatics, Wenzhou Medical College, Wenzhou, China; 4 Key Laboratory of Pathogenic Microbiology and Immunology, Institute of Microbiology, Chinese Academy of Sciences, Beijing, China; 5 Department of Microbiology and Immunology, Indiana University School of Medicine, Indianapolis, Indiana, United States of America; Mahidol University, Thailand

## Abstract

**Background:**

*Leptospira interrogans* is the major causative agent of leptospirosis. Phagocytosis plays important roles in the innate immune responses to *L. interrogans* infection, and *L. interrogans* can evade the killing of phagocytes. However, little is known about the adaptation of *L. interrogans* during this process.

**Methodology/Principal Findings:**

To better understand the interaction of pathogenic *Leptospira* and innate immunity, we employed microarray and comparative genomics analyzing the responses of *L. interrogans* to macrophage-derived cells. During this process, *L. interrogans* altered expressions of many genes involved in carbohydrate and lipid metabolism, energy production, signal transduction, transcription and translation, oxygen tolerance, and outer membrane proteins. Among them, the catalase gene expression was significantly up-regulated, suggesting it may contribute to resisting the oxidative pressure of the macrophages. The expressions of several major outer membrane protein (OMP) genes (e.g., *ompL1*, *lipL32*, *lipL41*, *lipL48* and *ompL47*) were dramatically down-regulated (10–50 folds), consistent with previous observations that the major OMPs are differentially regulated *in vivo*. The persistent down-regulations of these major OMPs were validated by immunoblotting. Furthermore, to gain initial insight into the gene regulation mechanisms in *L. interrogans*, we re-defined the transcription factors (TFs) in the genome and identified the major OmpR TF gene (LB333) that is concurrently regulated with the major OMP genes, suggesting a potential role of LB333 in OMPs regulation.

**Conclusions/Significance:**

This is the first report on global responses of pathogenic *Leptospira* to innate immunity, which revealed that the down-regulation of the major OMPs may be an immune evasion strategy of *L. interrogans*, and a putative TF may be involved in governing these down-regulations. Alterations of the leptospiral OMPs up interaction with host antigen-presenting cells (APCs) provide critical information for selection of vaccine candidates. In addition, genome-wide annotation and comparative analysis of TFs set a foundation for further studying regulatory networks in *Leptospira* spp.

## Author Summary

Leptospirosis is an important tropical disease around the world, particularly in humid tropical and subtropical countries. As a major pathogen of this disease, *Leptospira interrogans* can be shed from the urine of reservoir hosts, survive in soil and water, and infect humans through broken skin or mucous membranes. Recently, host adaptability and immune evasion of *L. interrogans* to host innate immunity was partially elucidated in infection or animal models. A better understanding of the molecular mechanisms of *L. interrogans* in response to host innate immunity is required to learn the nature of early leptospirosis. This study focused on the transcriptome of *L. interrogans* during host immune cells interaction. Significant changes in energy metabolism, oxygen tolerance and outer membrane protein profile were identified as potential immune evasion strategies by pathogenic *Leptospira* during the early stage of infection. The major outer membrane proteins (OMPs) of *L. interrogans* may be regulated by the major OmpR specific transcription factor (LB333). These results provide a foundation for further studying the pathogenesis of leptospirosis, as well as identifying gene regulatory networks in *Leptospira* spp.

## Introduction

Leptospirosis, which is characterized by hemorrhage, diarrhea, jaundice, severe renal impairment, and aseptic meningitis, *etc.*, has emerged as a global zoonotic infectious disease in the past decade [1]. Several pathogenic *Leptospira* species cause infection, which include more than 15 genospecies and 230 serovars distributed geographically. Other free-living saprophytic *Leptospira* species, such as *Leptospira biflexa*, do not infect humans and animals. The pathogenic, saprophytic *Leptospira* and several other intermediate species all belong to the *Spirochaetes*, a unique phylum in eubacteria including other pathogens, such as *Borrelia burgdorferi* and *Treponema pallidum*. *Leptospira interrogans* is the most prevalent pathogenic *Leptospira* species which survives in natural environments and animal reservoir hosts, and infects humans through abrasions in the skin or mucous membrane. The main reservoir hosts of *L. interrogans* are wild rodents and domestic animals, which can persistently excrete *L. interrogans* through urine. The shed leptospiral cells can survive in moist soil and water for a long time before infecting a new host [2]. Therefore, *L. interrogans* adapts to diverse natural environments and evades host immune defense during infection to maintain transmission. This makes *L. interrogans* an important pathogen in understanding leptospirosis.

The genome sequences of *L. interrogans* (strain Lai 56601 and Fiocruz L1-130), pathogenic *Leptospira borgpetersenii* (strain L550 and JB197), and saprophytic *L. biflexa* (strain Patoc I Paris/Ames) have been released in the past few years [3], [4], [5], [6]. The genome size of *L. interrogans* (∼4.6M) is larger than those of *L. borgpetersenii* (∼3.9M) and *L. biflexa* (∼3.9M), which is consistent with the evidence that *L. interrogans* retained more genes from the common ancestor while acquiring exogenous genes during evolution. Comparative genomics have been preformed to identify potential virulence genes in *L. interrogans* [7]. However, few virulence factors have been experimentally confirmed due to the lack of efficient methods for genetic manipulation of pathogenic *Leptospira* [8]. In addition, many of the putative functional genes are in multicopies or families with high degree of redundancy, which further hampers virulence determinants using genetic approaches and molecular Koch's postulate. For example, two major outer membrane protein genes, *ligB* [9] and *lipL32* [10], which are highly conserved in pathogenic *Leptospira* and absent in non-pathogenic *L. biflexa*, have been inactivated in *L. interrogans* and verified to be dispensable for infection.

In comparison to the other pathogenic spirochetes, *L. interrogans* encodes more putative signal transduction and transcriptional regulation genes [11]. Several global gene expression studies have elucidated the transcriptional responses of *L. interrogans* to temperature, osmolarity, and host serum [12], [13], [14], [15]. Among these factors, osmotic stress was identified as a key signal affecting the leptospiral transcriptome. However, these microarray analyses identified few genes whose expression has been shown to be differentially regulated during mammalian infection by proteomics and other approaches [16], [17], [18]. In particular, several major OMPs genes (e.g., *lipL32*, *qlp42* and *loa22*) are differentially regulated *in vivo*. This is likely due to the environmental factors *in vitro* are not the major signals *Leptospira* senses during mammalian infection. Therefore, global analysis of leptospiral gene expression in animal or infection models are vital to identify differentially regulated genes relevant to pathogenesis.

Co-cultivation of pathogenic *Leptospira* with host immune cells is widely used as an infection model to study leptospirosis [19], [20]. Although pathogenic *Leptospira* is not considered a typical intracellular pathogen, recent studies showed that pathogenic *Leptospira* can attach, invade, and induce apoptosis of mammalian macrophages, and escape host innate immunity during the early stage of infection [21], [22]. In addition, our study demonstrated differential survivability of *L. interrogans* within murine or human macrophages, which may contribute to the different severity between the mild chronic infection in reservoir animals and the acute lethal infection in humans [23]. Rapid uptake of *L. interrogans* by phagocytes were also verified by the naive zebrafish embryos model, suggesting that phagocytosis may be a key defense mechanism during the early stage of infection [24]. In this study, we performed microarray analysis on leptospiral gene expression in response to innate immune cells of murine and human origin. We found a dramatic influence of *L. interrogans* gene expression by host macrophage interaction, including genes of the major OMPs. A bioinformatic approach was used to determine regulators responsible for differential gene expression. This approach identified a putative OmpR transcription factor, which may be involved in the regulation of major OMP genes.

## Materials and Methods

### Bacterial strain

*L. interrogans* Serovar Lai Strain Lai 56601 was obtained from the National Institute for the Control of Pharmaceutical and Biological Products, Beijing, China. For microarray hybridization purpose, a single colony was picked from the EMJH [25], [26] plate (1% agar) and verified by 16S rDNA-specific and *gyrB1* (DNA gyrase subunit B1 gene)-specific primer PCR and gene sequencing. The virulence of the *L. interrogans* isolate was restored by passage through Dunkin-Hartley ICO: DH (Poc) guinea pigs (10–12days old, weighing 120–150g each) before infection. As an *in vitro* control design, the isolate was cultured in liquid EMJH medium for 5 passages and named E0 sample after the EMJH medium. The culture condition of each passage was growth in 200ml liquid EMJH media at 28°C under aerobic conditions for 120 h to reach exponential growth phase. Three biological replicates (E0-1/2/3) were used for microarray purpose. Before sample collection, one volume of bacterial culture was mixed with a one-tenth volume of ice-cold phenol/EtOH stop solution [10% water-saturated phenol (pH<7.0) in ethanol] and chilled rapidly [27]. Leptospiral cells were harvested by centrifugation at 8,000 g, 4°C for 15 min. All animals were handled in strict accordance with good animal practice as defined by the relevant national and/or local animal welfare bodies, and all animal work was approved by the Animal Ethics Review Committee of Zhejiang University.

### Host cell lines

Murine monocyte-macrophage-like cell line J774A.1 and human acute monocytic leukemia cell line THP-1 were obtained from American Type Culture Collection (Manassas, VA) and grown in RPMI 1640 medium (Invitrogen, Carlsbad, CA) supplemented with 10% (V/V) heat-inactivated fetal calf serum (FCS, Gibco/Invitrogen, Carlsbad, CA) with antibiotic, in a humidified 5% CO_2_ atmosphere at 37°C. The suspended THP-1 cells were treated with 5 nM phorbol myristate acetate (PMA; Sigma-Aldrich St. Louis, MO) for 24h. After differentiation, the cells were washed three times with sterilized PBS buffer, and rested for 24h in new cell medium to ensure that they reverted to a resting phenotype before infection. All cells were cultured in 225 cm^2^ tissue culture flasks (Corning. Inc., Big Flats, NY) and the cell numbers were counted using haemocytometer.

### Infection models

**Figure 1:**
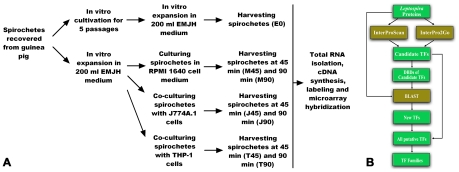
Schematic representation of the macrophage infection models (A) and search tactics of specific transcription factors from the leptospiral genomes (B).

The cultured mammalian cells were washed three times with sterilized PBS buffer to remove antibiotic, fresh media without antibiotics were added, and cultured for an additional 12 h before infection. Leptospiral cells were harvested by centrifugation at 8, 000 g, 20°C for 15 min, and washed three times with sterilized PBS buffer. The leptospiral pellets were re-suspended in 37°C RPMI 1640 medium with 10% (V/V) heat-inactivated FCS and the bacterial numbers were counted with a Petroff-Hausser counting chamber (Fisher Scientifics, Houston, Texas). Then 10 ml of leptospiral suspension (10^9^) were added into 10^7^ macrophage cells (bacteria∶cell = 100∶1) and incubated in 5% CO_2_ at 37°C. These co-cultured *L. interrogans* samples were defined as J (J774A.1) and T (THP-1) samples respectively after the names of the mammalian cell lines. In order to evaluate the impact of mammalian cell culture medium on *L. interrogans*, RPMI 1640 medium controls [RPMI 1640 medium with 10% (V/V) heat-inactivated FCS] were introduced into experiments as M (RPMI 1640) samples. That is, *L. interrogans* grew in RPMI 1640 medium with 10% (V/V) heat-inactivated FCS which had been deposited in 5% CO_2_ at 37°C for 12 h beforehand. Three biological replicates were designed for each sample for microarray purpose. To guarantee the integrity of the total RNA, the survival of the *L. interrogans* samplings drawn from all above-mentioned infection models were verified by darkfield microscope analysis (400×). Then the co-cultured *L. interrogans* samples (J, T and M) were RNA-stabilized and collected at 45 min when *L. interrogans* began to attach the host cells, or at 90min when the attachment rate reached the stable level [28]. In detail, the attached leptosiral cells were gathered by washing the macrophage cells twice with sterilized PBS at the time-points of 45 min and 90 min respectively. The collections were mixed with a one-tenth volume of ice-cold stop solution and chilled immediately. Then, the mixtures were centrifuged at 1, 000 g for 5 min at 4°C to exclude the pellets of J774A.1 and THP-1 cells. The supernatants were centrifuged at 8, 000 g, 4°C for 15 min to collect the leptospiral pellets. This RNA stabilization procedure is essential for microarray analysis because of the short life time of leptospiral total RNA (**Figure 1A**).

### RNA purification and ds cDNA synthesis

Leptospiral total RNA was extracted using TRIzol reagent (Invitrogen, Carlsbad, CA), then purified by RNeasy Mini Kit (QIAGEN, Hilden, Germany) with on-column DNase digestion (QIAGEN, Hilden, Germany) according to the RNeasy Mini handbook. RNA quantity and integrity was determined using the RNA 6000 Nano Laboratory-on-a-Chip kit and the Bioanalyzer 2100 (Agilent Technologies, Palo Alto, CA). For each sample, about 10 µg of total RNA was mixed with 600 ng of random hexamer primers (TaKaRa, Otsu, Japan) and denatured at 65°C for 5 min. Then the first strand cDNA was synthesized using 2 µl (400 U) SuperScript III reverse transcriptase (Invitrogen, Carlsbad, CA) according to the protocol recommended by the manufacturer. The double strand cDNA (ds cDNA) sample was synthesized using the 2nd Strand Synthesis section of the M-MLV RTase cDNA Synthesis Kit (TaKaRa, Otsu, Japan) according to the manufacturer's instructions. Following RNase H (Invitrogen, Carlsbad, CA) and RNase A (Ambion, Austin, TX) digestion for 1 h, ds cDNA sample was purified with the QIAquick PCR Purification Kit (QIAGEN, Hilden, Germany) according to the QIAquick Spin handbook.

### Microarrays and hybridization

The *L. interrogans* Serovar Lai Strain Lai 56601-specific high-density, photolithography-based, mono-plex DNA microarray chip was designed and produced by Roche NimbleGen, Inc. Each slide consisted of a total of 385,000 oligonucleotide probes (60-mer each probe) which covered all predicted 4,727 ORFs of the whole genome (NC_004342 and NC_004343). In average, sixteen probes were designed for each ORF, which is one of the strength of this technology. Each probe pair consisted of a sequences matched to the ORF, and another adjacent sequence harbored mismatched bases for the determination of background and cross-hybridization. Note that the original annotation for *L. interrogans* Serovar Lai Strain Lai 56601 included more than 900 putative small ORFs (less than 150 bp). In contrast, the homologs of these small ORFs were not included in the later genome annotation for other 5 *Leptospira* strains. However, we found that some of these putative ORFs had very high level of expression (Data not shown). Thus, these small ORFs were included in our microarray analysis. For each hybridization, 1 µg of ds cDNA was labeled with Cy3-9mer Primers (TriLink Biotechnologies, San Diego, CA) using the Klenow fragment (New England Biolabs, Beverly, MA) exo-extending reaction. 1.5 µg of Labeled cDNA sample was individually hybridized to the microarray using the MAUI hybridization system from Roche NimbleGen, then washed and dried according to the Roche NimbleGen standard procedure.

### Data extraction and statistical analysis

The microarrays were scanned using the Axon GenePix 4000B microarray scanner at 5-µm resolution. The data were extracted using Roche NimbleGen NimbleScan™ software and an algorithm (courtesy of Y. Qiu, University of Wisconsin School of Medicine) was applied to obtain a single measurement of signal intensity for each ORF. Data were normalized using the quantile normalization method and the total signal intensity of a given ORF was converted to estimates of transcript abundance by using the robust multiarray average (RMA) procedure. For all microarrays, a *P* value for each ORF was calculated by a two-tailed Welch's unpaired *t* test comparison of the five microarray replicates for each sampling condition. The fold changes of an ORF between two relevant sampling conditions were calculated as the division value of average signal intensity of 3 biological replicates: average J, T or M sample signal intensity at a certain time-point/average E sample signal intensity. Only fold changes of at least ±2 and *P*≤0.05 were considered significant and included in this report.

### Validation of microarray data by quantitative real-time RT-PCR

Primers for six randomly selected *L. interrogans* Serovar Lai Strain Lai 56601 genes (**Table S1**) were designed with Primer Premier software version 5 (Premier Biosoft International, Palo Alto, CA). New batches (in triplicate) of bacterial RNA were used in real-time RT-PCR. RT reaction mixtures contained 1 µg of total RNA, 300 ng of random hexamer primers (TaKaRa, Otsu, Japan), 0.5 µl (100 U) of Superscript III (Invitrogen, Carlsbad, CA) reverse transcriptase, and 500 µM concentrations each of dATP, dCTP, dGTP, and dTTP. After denaturation on 65°C for 5 min, the samples were incubated at 50°C for 1 h, followed by 10 min at 70°C to synthesize the first strand cDNA. 50 ng of cDNA were mixed with 12.5 µl of 2×SYBR Green PCR Master Mix (Applied Biosystems, Foster City, CA). Assays were performed in triplicate with the ABI PRISM model 7500 sequence detection instrument. Amplicon quantification in real-time RT-PCR was performed by comparison with gene-specific standard curves constructed form known concentrations of each purified amplicon. The melting curve analysis was also used to evaluate that the accumulation of SYBR Green-bound DNA was gene-specific.

### Extraction of total proteins and outer membrane proteins

The *Leptospira* pellets were harvested from infection models at 1h, 2h, and 4h according to the method described previously in this report. Total leptospiral protein was extracted with Triton X-100. For each sample, the leptospiral pellet was washed twice in 0.5 ml PBS-5mM MgCl_2_ and resuspended in 0.5 ml bacteria lysis buffer which was composed of 50 mM Tris-HCl (pH8.5), 2 mM EDTA, 100 mM NaCl, 0.5% Triton X-100 (Calbiochem, La Jolla, CA), 100 µg/ml lysozyme (Sigma-Aldrich St. Louis, MO) and 1µl/ml protease inhibitor PMSF (Sigma-Aldrich St. Louis, MO). The suspension sample was subjected to three cycles of freezing, thawing and tip sonication, following by centrifugation at 10,000g for 10 min to exclude the indissoluble fragments. The soluble supernatant was dialyzed by 1% SDS and tested by SDS-PAGE electrophoresis. The leptospiral OMP samples were extracted by solubilization with 1% Triton X-114 (Calbiochem, La Jolla, CA) according to the method reported previously [29].

### Recombinant proteins expression and immunization procedure

The full-length PCR products of the genes of LipL32 (LA2637), LipL41 (LA0616), OmpA (LB328), OmpL1 (LA3138), Mce (LA2055), FliH (LA2589), FliI (LA2592), FliY (LA2613), FliN (LA2069) were amplified from *L. interrogans* Serovar Lai Strain 56601 chromosomal DNA using gene-specific primers, inserted into pGEM-T easy vectors (Promega, Madison, WI) and verified by gene-sequencing. Then the target genes were double-digested and inserted into pET-42a (+) vectors (Merck Novagen, Nottingham, UK), verified by sequencing and expressed in *E. coli* Rosetta™ strain (Merck Novagen, Nottingham, UK) as N-terminal 6×His-tagged recombinant proteins. Recombinant proteins were purified by Ni-NTA agarose column (QIAGEN, Hilden, Germany), mixed with complete Freund's adjuvant (Sigma-Aldrich St. Louis, MO), and used to immunize SPF New Zealand rabbits at days 1, 15, 30, 45. On days 55, the blood samples were taken from the rabbits' hearts and the effect of antisera was calculated by ELISA tests. The antisera were diluted into appropriate solutions (1∶800 for LipL32/41, OmpA, OmpL1, FliY and FliN, 1∶400 for Mce, FliH, and FliI), and used in the following Western blotting analysis.

### Verification of protein changes by Western blotting

Protein concentration was estimated by BCA protein assay (Pierce, Rockford, IL). For leptospiral lipoproteins (LipL32, LipL41) and outer membrane protein A (OmpA), the OMP samples were used in Western blotting analysis. For transmembrane protein (OmpL1), flagellar components (FliH, FliI, FliY and FliN) and intracelluar protein (Mce), the total protein samples were used. Equivalent amounts of protein (1 µg) were separated by 1-D electrophoresis with 10% SDS-PAGE, and electrotransferred onto polyvinylidene difluoride membranes (PVDF membranes, Millipore, Billerica, MA) using a Trans-Blot Semi-Dry Transfer Cell (Bio-Rad, Hercules, CA). The membranes were blocked in TBST [20 mM Tris (pH 7.6), 137 mM NaCl, 0.1% Tween 20] containing 5% non-fat milk and probed with corresponding antibodies (1∶2000) respectively overnight at 4°C. Then, the membranes were incubated with peroxidase-conjugated anti-rabbit immunoglobulin G (Jackson ImmunoResearch Laboratories Inc., PA) for 2 h at room temperature and visualized on X-ray film using enhanced chemiluminescence reagents (Millipore, Billerica, MA). The band intensities were estimated by densitometric scanning using the Gel Doc 2000 system (Bio-Rad, Redmond, WA) and Quantity One software. Data shown are from three independent experiments.

### Genome annotation, cluster and pathway analysis of microarray data

To supply new functional annotation of the *L. interrogans* Serovar Lai Strain Lai 56601 genome, assignment of putative functions was performed by means of a combination of BLAST-based and HMM-based searches of the KEGG gene database and InterPro protein domain database. Hierarchical cluster analysis of microarray data was preformed by the Cluster 3.0 software and visualized by the TreeView software [30]. Several similarity matrices, including correlation matrices [Correlation (uncentered), Absolute Correlation (uncentered), Spearman Rank Correlation, Kendall's tau] and distance matrices (Euclidean distance and City-block distance) were used in cluster analysis to define the significantly changeable subclades. The KEGG pathway was introduced to analyze the changing magnitude in each biological pathway by gathering statistics from the significant gene expression changes. The proportion of regulated genes in the 14 leptospiral KEGG pathway was calculated and displayed by Microsoft Excel diagram.

### Discovery and evolutionary analysis of transcription factors

Candidate TFs were collected by InterProScan program (http://www.ebi.ac.uk/Tools/InterProScan/) and the Gene Ontology terms were obtained from the InterProScan results using InterPro2GO (http://www.geneontology.org/external2go/interpro2go) [31]. Then, the DNA binding domains (DBDs) of the candidate TFs were used in further BLAST search against all *Leptospira* proteins to identify new TFs. All putative TFs were classified into different families according to categories of their DBDs. This strategy helped us to find new TFs that had not been defined before (**Figure 1B**). Finally, phylogenetic analyses of the whole TF sequences were performed using Neighbor-Joining method of MEGA 4.0 software to reveal the evolutionary relationship of the putative TFs [32].

## Results and Discussion

### Annotation of previous defined hypothetical ORFs

In order to conduct genome-wide transcriptional analyses of *L. interrogans*, we first performed an updated annotation using BLAST and InterProScan tools with up-to-date databases. Specifically, we focused on annotating the hypothetical ORFs, which comprises about 40% of total ORFs of the genome of *L. interrogans* Serovar Lai Strain Lai 56601 [3]. This annotation allowed us to assign putative functions to 375 hypothetical proteins (**Table S2**), and identified several functionally important homologs missing in the previously annotated genome. These included a ferrous iron transporter, outer membrane lipoprotein carrier protein LolA, phospholipase C, the SecE subunit for protein translocation complex, a cell wall hydrolase, and the flagellar hook-length control protein chromogranin. In addition, several ORFs involved in host-pathogen interaction were identified, including a haem oxygenase-like protein, type-III fibronectin, hemopexin, a prevent-host-death protein, caspase catalytic proteins, a nitrilase/cyanide hydratase, ricin B lectin, and cadherin-like protein.

### Microarray experimental design

To study global transcriptional responses of *L. interrogans* upon interaction with the host innate immune system, we chose two different mammalian cell lines: murine monocyte-macrophage-like cell line J774A.1 and human acute monocytic leukemia cell line THP-1. This represented interaction with macrophage of either natural mammalian reservoir or human host. Briefly, a clone of *L. interrogans* Serovar Lai Strain Lai 56601 was used to infect a guinea pig and subsequently recovered from kidney tissue. Isolated infectious spirochetes were split to two samples. One sample was kept under *in vitro* cultivation and passage in EMJH medium for 5 passages (approximately one month) then subjected to RNA extraction and defined as *in vitro* cultivation sample (sample E0). The other sample was divided into three groups and co-cultured with cell lines J774A.1, THP-1, or RPMI 1640 medium with 10% heat-inactivated FCS designated as samples J, T, and M, respectively. After 45 or 90 minutes, spirochetes were harvested, subjected to RNA extraction, and cDNA synthesis (**Figure 1A**). Each cDNA sample was labeled and used for a single hybridization. Average signal intensity of 3 biological replicates was calculated as a valid data for each sample (E0, J45, J90, T45, T90, M45 or M90). The transcriptional fold-changes of ORFs in J, T or M samples were calculated relative to sample E0. Analysis of differentially expressed genes were confined to those with changes ≥2-fold (*P*≤0.05). The microarray raw data are available at http://cibex.nig.ac.jp/ under CIBEX accession no. CBX129.

### Global transcriptomic analysis

**Figure 2:**
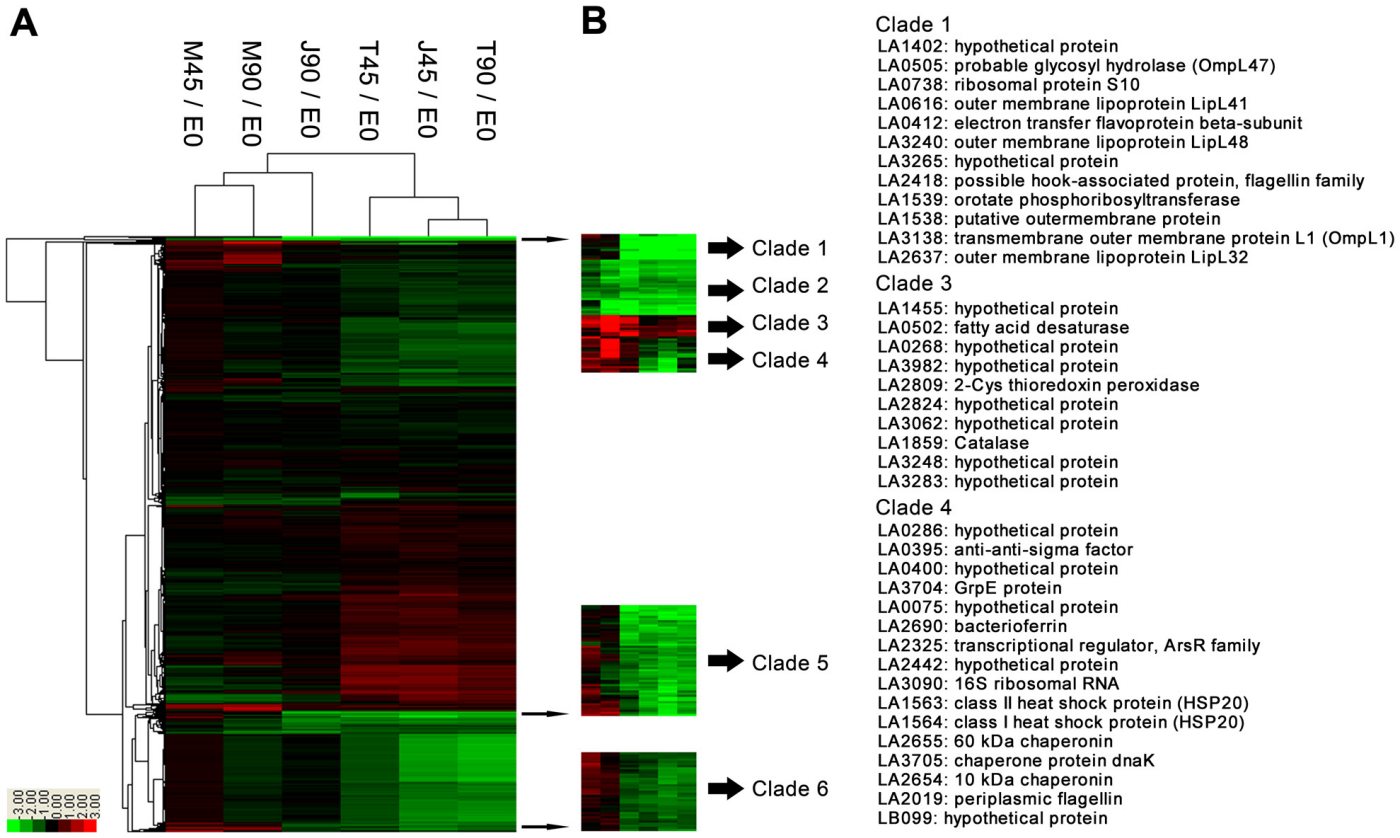
Genome-wide transcriptional changes of the *L. interrogans* Serovar Lai Strain Lai 56601 in the infection models. Cluster analysis (Euclidean distance) revealed several distinct subclades in the whole transcriptomics (**A**). The subgroup of most highly down-regulated genes was defined as Clade 1, which included several major outer membrane protein genes, such as *ompL47* (LA0505), *lipL41* (LA0616), *lipL48* (LA3240), o*mpL1* (LA3138), and *lipL32* (LA2637), *etc*. The most significantly up-regulated genes were included in Clade 3. The Clades 2,4,5 and 6 included the moderately down-regulated genes (**B**).

Based on the correlation and distance similarity matrices of Cluster 3.0 software, *L. interrogans* genes differentially expressed upon interaction with macrophages were grouped into several clades (**Figure 2A**). Clades of up-regulation and down-regulation of gene expression occurred mainly in comparison between the *in vitro* cultivated spirochetes and bacteria that interacted with either mouse-derived macrophage or human macrophage cell lines, not in comparison between the *in vitro* cultivated spirochetes and bacteria in RPMI 1640 medium controls. In other words, incubation of spirochetes with the cell culture medium alone had little influence in gene expression (M45/E0 and M90/E0). In addition, the cluster analysis showed that T45/E0, T90/E0 and J45/E0 were closely related, whereas J90/E0 was similar to M45/E0 and M90/E0, both in correlation matrices and in distance matrices. This suggested a more transient change of gene expression profile in *L. interrogans* upon interaction with murine macrophages (J90/E0) than with human-derived macrophage cells (T90/E0). About 65% of down-regulated genes and 45% up-regulated genes were hypothetical protein genes, which was consistent with previous transcriptomic results that these genes are often regulated in different environments [12], [14].

Cluster analysis revealed a distinct clade, Clade 1, containing a set of highly down-regulated genes (30 to 50-fold) (**Figure 2B**). Intriguingly, some of the well-studied major OMP and lipoprotein genes fell into Clade 1, including LA0505 (*ompL47*) [33], LA0616 (*lipL41*) [34], LA3240 (*lipL48*) [35], LA3138 (*ompL1*) [36] and LA2637 (*lipL32*) [17], [18], [29], . The down-regulation of Clade 1 was mainly due to the interaction with host macrophage, which was different from the moderately down-regulated Clade 2. The Clade 2 differed from Clade 1 because its moderate down-regulation was also observed in the RPMI 1640 medium controls. Another distinct clade, Clade 3, included a set of highly up-regulated genes (3 to 7-fold) which may contributed to oxygen tolerance, such as the fatty acid desaturase gene (LA0502), the 2-Cys thioredoxin peroxidase gene (LA2809) and the catalase gene (LA1859). These genes were regulated not only upon interaction with macrophages, but also in RPMI 1640 medium controls. Interestingly, the Clade 4 included several chaperone and heat shock protein genes, such as GrpE gene (LA3704), HSP20 genes (LA1563 and LA1564), 60 kDa chaperonin gene (LA2655), 10 kDa chaperonin gene (LA2654), and chaperone protein DnaK gene (LA3705), *etc*. These genes were persistently up-regulated in RPMI 1640 medium controls (M45/E0, M90/E0) and maybe due to the elevated temperature [12], but transiently down-regulated upon interaction with host macrophage cells at the 45 min time-point (T45/E0, J45/E0). At the 90 min time-point, *L. interrogans* moderately up-regulated this clade upon interaction with murine J774A.1 cell lines (J90/E0), but remained down-regulated upon interaction with the human THP-1 cell lines (T90/E0). The implication and mechanism of this differential regulation of Clade 4 upon interaction with murine vs. human cell lines are still unclear. In addition, two moderately down-regulated clades, Clade 5 and Clade 6, were defined for discussions in the corresponding section below.

**Figure 3:**
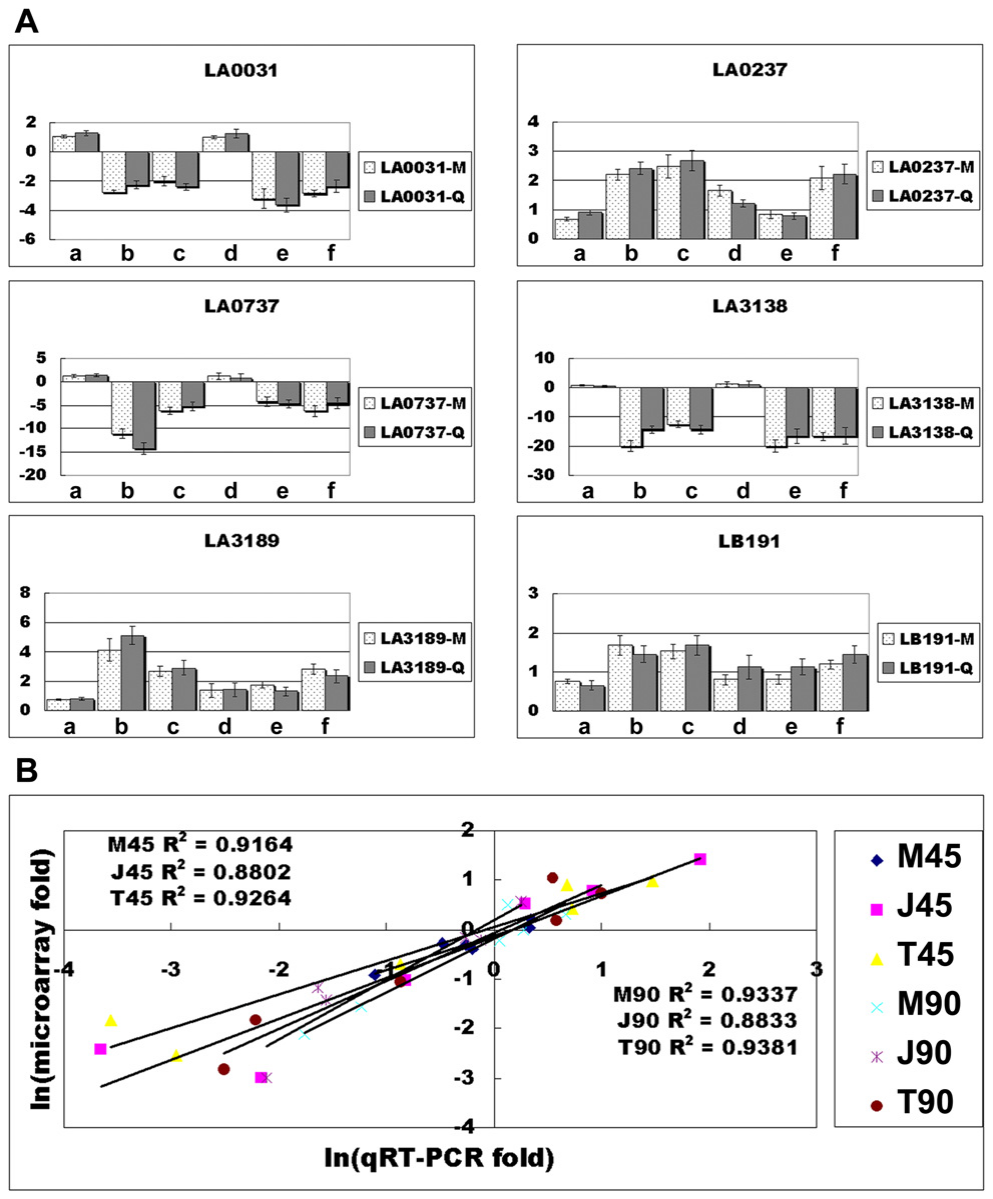
Validation of microarray data using quantitative real-time RT-PCR. The transcriptional levels for the randomly selected 6 genes (Table S1) were determined by quantitative real-time RT-PCR using new batch of RNA samples (**A**). M: the mRNA change folds from normalized microarray data; Q: the mRNA change folds from normalized qRT-PCR data; a, b, c, d, e, and f: the mRNA change folds of M45, J45, T45, M90, J90 and T90. No PCR amplification was detected in negative controls. The quantitative real-time RT-PCR values were plotted against the microarray data values. The high correlation coefficient values (R^2^) indicated that the microarray signal represented by multiple oligonucleotide probes was valid for transcriptomics research (**B**).

**Figure 4:**
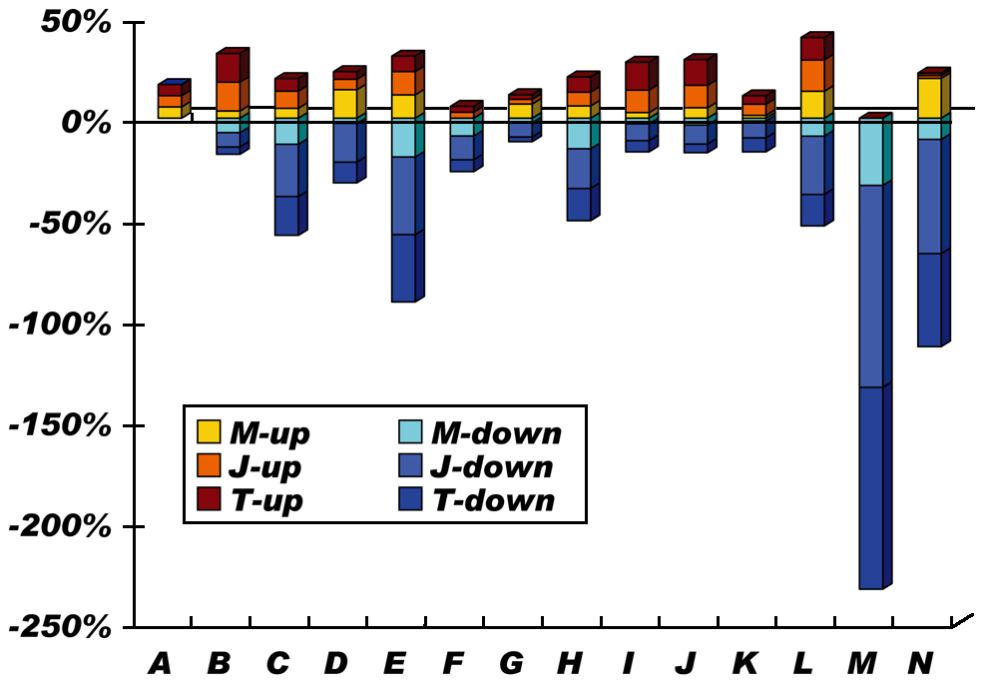
Statistic analysis of the leptospiral transcriptional regulation based on KEGG pathway. The percentage of differentially regulated genes was calculated by dividing the number of up-regulated or down-regulated genes by the total number of genes in each category, respectively. A, Biosynthesis of Polyketides and Nonribosomal Peptides (9 genes); B, Biosynthesis of Secondary Metabolites (28 genes); C, Carbohydrate Metabolism (224 genes); D, Cell Motility (78 genes); E, Energy Metabolism (78 genes); F, Folding, Sorting and Degradation (34 genes); G, Glycan Biosynthesis and Metabolism (43 genes); H, Lipid Metabolism (132 genes); I, Membrane Transport (36 genes); J, Metabolism of Cofactors and Vitamins (117 genes); K, Replication and Repair (72 genes); L, Signal Transduction (45 genes); M, Transcription (3 genes); N, Translation (76 genes). M-up, J-up, and T-up: the percentages of up-regulated genes in M, J and T samples; M-down, J-down, and T-down: the percentages of down-regulated genes in M, J and T samples. A gene regulated either at a time-point or at two time-points was included in this statistics analysis. If a gene was up-regulated at a time-point but down-regulated at another time-point, it was included both in up-regulation and in down-regulation.

Validation of the microarray data by qRT-PCR was shown in **Figure 3**. To evaluate the alterations in leptospiral biological pathways, proportions of up-regulated and down-regulated genes in KEGG pathways were calculated (**Figure 4**). Transcription and translation systems, carbohydrate, energy and lipid metabolism, and signal transduction systems exhibited significant down-regulation patterns, while biosynthesis of secondary metabolites, membrane transport and metabolism of cofactors and vitamins showed up-regulation patterns in our infection models. In addition, several important KEGG sub-pathways were significantly altered. Most genes of the citric acid cycle (TCA cycle), flagellar assembly, oxidative phosphorylation, fatty acid metabolism and ribosome synthesis were down-regulated, while most genes of starch and sucrose metabolism, porphyrin metabolism, and two-component systems were up-regulated (Data not shown). Categories exhibiting significant regulation are discussed below in detail.

### Energy, carbohydrate, and lipid metabolism

Most of the genes involved in this category were down-regulated upon interaction with macrophages. First, genes involved in oxidative phosphorylation were down-regulated. These changes included a significant down-regulation (3–6 folds) of a putative operon (LA0242-0244, included in down-regulated Clade 5) encoding cytochrome caa3 oxidase subunit II, cytochrome C oxidase polypeptide I and cytochrome C oxidase polypeptide III (**Table S2**). Previous reports showed that the leptospiral cytochrome C oxidase polypeptide III is replaced by an alternative subunit that has weak oxygen affinity, which contributes to the low rate of oxidative phosphorylation in *L. interrogans* [4]. The reduced expression of this operon observed suggested that *L. interrogans* reduced its O_2_ requirement upon interaction with host cells. Furthermore, most genes of the citric acid cycle (TCA cycle) as well as a putative operon encoding the leptospiral F_0_F_1_ ATP synthetase subunits (LA2780-LA2782) [4], were significantly down-regulated upon interaction with macrophages.

*Leptospira* can not utilize glucose and other sugars as carbon source. Instead, it degrades long chain fatty acid through beta-oxidation for carbon and energy production. In fact, tween-80, not glucose, are essential ingredients of the semi-synthetic EMJH medium for *in vitro* growth of *Leptospira*. Consistent with this, recent comparative genomics analyses on six leptospiral genomes revealed that *Leptospira* has limited sugar transport system [3], [7]. As such, *L. interrogans* encodes more genes of the fatty acid metabolism genes than *Escherichia coli* K12. There are three homologs of long-chain-fatty-acid CoA ligase (LA0106, LA2177, and LA2309), which are the rate-limiting enzymes for fatty acid degradation [3]. Our microarray data revealed that expression of LA0106 and LA2309 were down-regulated (2–8 folds) during interaction with host cells. In addition, one of glycerol utilizing genes, LA0587, encoding lactonizing lipase, was dramatically down-regulated (4–9 folds). These results are consistent with down-regulation of its oxidative metabolism pathways described above.

In addition to the general trend of gene down-regulation, few genes in this category were up-regulated upon interaction with macrophages. Genes involved in nitrogen metabolism [2-nitropropane dioxygenase (LA2727), glutamate synthase (LB286)], were up-regulated. One of the genes in methane metabolism, the catalase gene (LA1859), was also significantly up-regulated 4–7 fold persistently. Since catalase is also involved in tryptophan metabolism and oxidative resistance, it remains to be determined the exact role the catalase plays during the initial stage of leptospiral infection. In addition, three genes in starch and sucrose metabolism [CDP-glucose 4,6-dehydratase (LA1632), alpha-glucosidase II (LA2944), glucose-6-phosphate isomerase (LA3888)] and three genes in glycolysis/gluconeogenesis [phosphoglyceromutase (LA0439), dihydrolipoamide dehydrogenase (LA2115), a probable alcohol dehydrogenase (LA2361)], were up-regulated. *L. interrogans* Strain Lai 56601 possesses the genes involved in the biosynthesis of unsaturated fatty acids pathway (KEGG pathway: lil01040), which suggests that this strain may synthesize some unsaturated fatty acids *de novo*. In contrary, *B. burgdorferi* has no unsaturated fatty acids biosynthesis genes, and instead scavenges polyunsaturated fatty acids from BSK II growth medium or hosts [40]. The putative rate-limiting enzyme in this pathway, omega-6 fatty acid desaturase gene (delta-12 desaturase gene, *desA*, LA0502), was dramatically up-regulated in RPMI 1640 medium controls (>10-fold). The relatively modest and late up-regulation of this gene upon interaction with macrophages may reflect the culturing conditions and micro-environments, such as elevated temperature and osmolarity [12], [15]. Considering that the RPMI 1640 medium controls and host cells were all eutrophic in unsaturated fatty acids, the implication and mechanism of the up-regulations of *desA* were still unclear.

### Oxygen tolerance and DNA repair

*L. interrogans* must evade oxidative killing mediated by host cells including macrophages. However, the *L. interrogans* genome has only few predicted genes involved in resistance to oxidative stress and reactive oxygen species (ROS). All four pathogenic leptospiral genomes lack homologues of *fqg*, *nfo*, *nei* or superoxide dismutase (*sod*) [3], [5]. *L. interrogans* Strain Lai 56601 has glutathione peroxidase genes (LA1007, LA4299) and thiol peroxidase gene (LA0862), but their level of expression were very low and did not have significant change upon interaction with macrophages. Pathogenic *Leptospira* also have cytochrome C oxidase genes, which may be involved in protection from O_2_ stress. Our microarray results showed a significant down-regulation of these genes (LA0242-0244), suggesting they may not be important for resistance to oxidative killing in our models.

Catalase is one of the proteins that plays an important role in resisting oxidative killing by phagocytes [41]. Both pathogenic and non-pathogenic *L. biflexa* have catalase genes in their genomes, but they are not homologs and belong to different enzyme groups: *L. interrogans* has a heme-containing *katE* homolog, whereas *L. biflexa* has a heme-containing dual functioning peroxidase/catalase *katG* homolog [42]. Our microarray result showed that expression of *katE* was very high and further up-regulated during interaction with host cells. However, this up-regulation appeared not to be the result of direct interaction with macrophages, but rather due to other host factors such as elevated temperature and mammalian serum, since *katE* gene expression was also increased in the M samples. This was consistent with previous reports that these factors can influence catalase expression in *Leptospira* [12], [15]. Interestingly, it was reported that non-pathogenic *Leptospira* is more susceptible to H_2_O_2_ killing *in vitro* [43], which suggests *katE* may play an important role in *Leptospira* infection.

In addition, the high expression and up-regulation of 2-Cys thioredoxin peroxidase gene (LA2809) indicated this gene may contribute to resisting oxidative stress. It was significantly up-regulated in RPMI 1640 medium (4–8 folds) and moderately up-regulated upon interaction with macrophages (2–3 folds). Cluster analysis revealed that this gene, the fatty acid desaturase gene and the catalase gene were assembled into a same clade, Clade 3 (**Figure 2B**). However, considering that it was not regulated by elevated temperature and host serum in previous studies [12], [15], the mechanism of its up-regulation may be different from those of the fatty acid desaturase and the catalase.

All six *Leptospira* genomes contain integrated DNA repair systems, such as the base-excision repair, the photo reactivate, and the SOS repair, *etc* [7]. Unexpectedly, no significant up-regulation of these genes in *L. interrogans* was found upon interaction with macrophages. The only major change was the down-regulation of the major recombinase A gene (*recA*, LA2179) in the homologous recombination pathway. The implication of this change remains unclear and suggested that *L. interrogans* experienced limited DNA damage under our conditions.

### Signal transduction, chemotaxis and motility

A distinct feature of *L. interrogans* relative to other spirochetal pathogens such as *B. burgdorferi* and *T. pallidum*, is that it has more two-component signal transduction systems (with more than 26 pairs of histidine kinases and response regulators). Regulation of two-component systems often occurs at the level of phosphorylation, not at the level of transcription. As expected, few obvious changes were observed in the microarray analysis for the histidine kinase and response regulator genes except that a two-component response regulator gene (LA2548) and a neighboring sensory transduction histidine kinase gene (LA2549) were moderately up-regulated temporarily at the 45 min time-point. In addition, other signal transduction related genes were differentially expressed. The acetyl-CoA acetyltransferase (LA0828) and the phosphate-binding protein PstS (LB297) were moderately down-regulated, while the operon including the potassium-transporting ATPase subunit A (*kdpA*, LA3112) and the potassium-transporting ATPase B chain (*kdpB*, LA3111) were moderately up-regulated at the 45 min time-point. The only change in the GGDEF sensory system was that the sensory box/GGDEF family protein (LA2931) was moderately up-regulated (**Table 1**).

**Table 1:** Category of leptospiral ORFs which were up-regulated at least 3-fold in infection models.

Clade ID	ORFID	M45/E0mean fold	J45/E0mean fold	T45/E0mean fold	M90/E0mean fold	J90/E0mean fold	T90/E0mean fold	Function and description of gene product
3	LA0268	5.3	1.56	1.11	16.71	6.18	1.95	hypothetical protein
	LA0273	0.79	2.65	3.11	1.24	1.4	2.07	lipoprotein releasing system transmembrane protein lolC
	LA0330	0.38	3.21	2.51	0.58	0.98	1.46	penicillin G acylase precursor (Penicillin G amidase, Penicillin G amidohydrolase)
	LA0356	0.64	3.01	1.6	0.88	1.45	2.56	hypothetical protein
	LA0366	0.58	2.25	3	1.19	1.33	2.15	phosphoserine aminotransferase (catalyzes the formation of 3-phosphonooxypyruvate and glutamate from O-phospho-L-serine and 2-oxoglutarate)
3	LA0502	3.36	0.96	0.95	14.96	5.84	1.98	fatty acid desaturase (Delta 12 desaturase)
	LA0625	0.8	2.03	3.21	1.27	0.74	1.61	DNA helicase RecQ
	LA0635	0.59	2.81	3.3	1.07	0.88	2.17	S-layer-like array protein
	LA0650	0.43	2.9	3.13	0.67	1.01	2.34	rhomboid family protein
	LA0662	0.72	2.26	3.08	0.84	0.8	1.55	chemotaxis motA protein
	LA0701	0.67	3.09	2.67	0.87	0.92	1.96	leucine-rich repeat containing protein
	LA0784	0.89	1.89	3.07	1.35	1.16	1.57	hypothetical protein
	LA0884	0.55	2.59	3.11	0.84	1.26	1.78	NADH dehydrogenase I, N subunit
	LA1122	0.6	3.13	3.09	0.92	1.22	1.81	putative outermembrane protein
	LA1334	0.63	2.39	3.81	0.83	1.15	1.72	putative oxidoreductase
	LA1854	0.59	3.03	2.5	0.79	1.04	2.86	hypothetical protein
3	LA1859	1.71	1.85	1.76	7.51	4.69	5.28	catalase
	LA1933	0.8	3	2.75	1.17	1.13	1.76	tetracycline resistance protein
	LA1937	0.43	2.64	3.07	0.53	1.14	3.12	predicted transcriptional regulator, copG family
	LA1944	0.49	3.41	4.27	0.54	1.64	2.17	putative lipoprotein
	LA1979	0.94	1.61	1.35	0.98	3.08	1.55	putative glycosyl transferase
	LA2032	0.41	2.56	2.6	0.48	1.08	3.02	predicted transcriptional regulator, copG family
	LA2156	0.6	2.63	3.04	0.97	1.56	1.97	aminotransferase
	LA2275	0.56	3.11	3.02	1.02	1.15	1.96	dedA protein
	LA2277	0.93	3.07	2.16	1.15	1.39	2.11	hypothetical protein
	LA2444	0.7	3.66	3.35	0.92	1.52	2.16	putative outermembrane protein
	LA2654	4.01	0.15	0.22	3.22	3.47	1.05	10 kDa chaperonin
	LA2659	0.98	2.77	3.59	1.43	1.51	2.32	hypothetical protein (Isopentenyl-diphosphate delta-isomerase, FMN-dependent; ATP-grasp fold)
3	LA2824	3.74	1.94	2.41	10.09	3.73	2.22	hypothetical protein (DoxX)
	LA2875	0.49	3.18	3.51	0.72	1.28	2.31	hypothetical protein
	LA2931	0.5	3.32	2.88	0.5	1.07	2.12	sensory box/GGDEF family protein
	LA3075	0.77	3.42	2.66	1.13	1.42	2.5	surface protein Lk90-like protein (LigC)
	LA3078	0.82	3.03	1.9	0.97	1.09	1.61	sterol desaturase family protein
	LA3189	0.73	4.11	2.68	1.36	1.76	2.84	hypothetical protein (CRISPR-associated protein, Cas6-related)
	LA3197	0.61	3.21	2.71	0.47	0.89	2.35	Type I restriction enzyme EcoR124II M protein
	LA3198	0.71	3.15	2.72	0.75	1.06	2.57	Type I restriction enzyme EcoprrI specificity protein
	LA3199	0.74	3.21	2.81	0.77	1.07	2.45	anticodon nuclease
	LA3216	0.65	2.51	3.06	1.08	1.59	2.15	octoprenyl-diphosphate synthase
3	LA3248	0.94	2.71	2.08	3.02	8.68	2.77	hypothetical protein
3	LA3283	0.93	2.69	1.86	2.91	8.6	2.74	hypothetical protein
	LA3287	0.64	3.84	2.28	0.8	1.11	2	hypothetical protein
	LA3353	0.89	1.27	1.08	0.91	4.73	1.15	hypothetical protein
	LA3414	0.51	3.3	3.2	0.9	1.86	3.1	hypothetical protein
	LA3574	0.65	2.47	3.08	0.74	0.99	1.79	flagellar protein FliL
	LA3630	0.7	2.59	3.28	0.91	1.36	2.54	probable transport ATP-binding protein msbA
	LA3726	0.82	3.19	2.49	0.96	1.39	2.66	hypothetical protein (Cadherin-like)
	LA3735	0.63	3.15	2.05	1.07	1.53	1.88	putative lipoprotein
	LA3736	0.77	3.33	2.43	0.86	1.23	1.3	TPR-repeat-containing proteins
	LA3801	0.94	3.21	2.9	0.92	1.1	2.02	glucosamine–fructose-6-phosphate aminotransferase (Hexosephosphate aminotransferase; D-fructose-6- phosphate amidotransferase) (GFAT) (L-glutamine-D-fructose-6-phosphate amidotransferase; Glucosamine-6-phosphate synthase)
3	LA3982	7.03	1.53	0.93	14.92	6.08	1.64	hypothetical protein
	LA4034	0.66	3.04	4.02	1.39	1.6	2.18	bacterial transferase family protein
	LA4046	1.28	2.68	3.29	0.89	1.23	1.91	hypothetical protein
	LA4128	0.64	3.03	2.62	0.98	1.96	2.27	putative lipoprotein
	LA4141	0.67	3.15	3.58	1.03	1.22	2.28	hypothetical protein
	LA4142	0.64	3.37	4.01	0.77	1.25	2.4	putative lipoprotein
	LA4148	0.87	3.42	2.54	1.09	1.85	2.67	hypothetical protein
	LB350	0.68	2.91	3.19	1.15	0.84	2.2	hypothetical protein

The ORFs up-regulated at least 3-fold in J or T samples were included in this table. The supplementary annotations generated in this study were showed in brackets. Clade ID: the clade ID for the significantly regulated ORF defined in genome-wide cluster analysis (Figure 2B).

**Figure 5:**
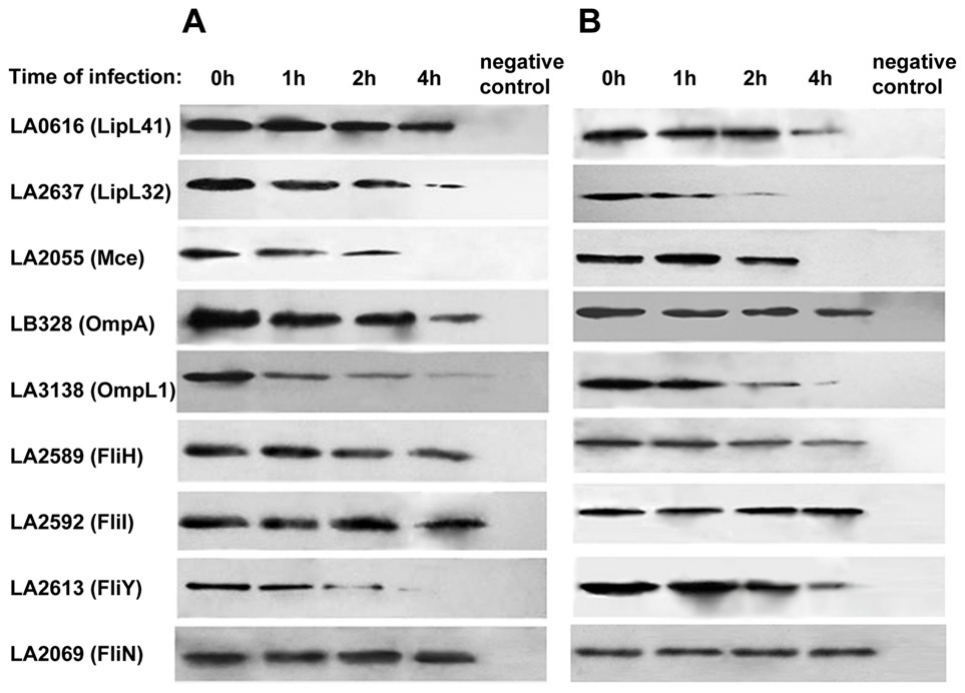
Verification of the leptospiral protein changes by Western blotting. The leptospiral samples at 1, 2 and 4 hour in the infection models [J774A.1 cell model (**A**) and THP-1 cell model (**B**)] were harvested for semi-quantitative protein assay. The protein expression levels of LipL41 (LA0616), LipL32 (LA2637), Mce (LA2055), OmpA (LB328), OmpL1 (LA3138), FliH (LA2589), FliI (LA2592), FliY (LA2613) and FliN (LA2069) were estimated by Western blotting band intensities.

Overall, genes involved in motility were down-regulated in *L. interrogans* upon interaction with macrophages. The major flagellin genes, *flaB1* (LA2017) and *flaB2* (LA2019), whose products had been confirmed by proteomics [39], [44], were down-regulated with a wide range (3–20 fold), suggesting reduced motility which is correlated with the down-regulation of energy generating metabolism during this process. Unlike the persistent down-regulation of the Clade 1, the expression of *flaB1* and *flaB2* were significantly down-regulated (15–20 fold) upon interaction with murine J774A.1 cell lines only at the 45 min time-point (J45/E0), but returned to their original E0 levels at the 90 min time-point (J90/E0). However, these transient down-regulations were not observed when *L. interrogans* interacted with human THP-1 cell lines. The down-regulation of *flaB1/2* in T samples was maintained less than 6-fold during the 90-min period. These different regulation patterns in J and T samples seemed to be related to the differences of invasive motility and immune evasion ability of *L. interrogans* infecting murine and human macrophage cells [23]. In addition, flagellar motor switch protein *fliY* gene (LA2613) was also down-regulated; this down-regulation was verified by immunoblotting analysis in this study (**Figure 5**). LA2418, which encodes another abundant flagellin protein, the hook-associated protein, showed 5 to 15-fold down-regulation. Consistent with the low motility and stable attachment of *L. interrogans* during the early stage of infection, a putative *cheY* gene (LA1253) which can interact with switch complex at the flagellar motor base to alter the rotating direction [45], was down-regulated at the time-point of 90 min in both T and J samples. The chemotaxis motA protein (LA0662) was up-regulated temporarily at the 45 min time-point (**Table 1**). The *flaB1/2* genes are typically used as an internal control for gene regulation in many studies. The observation of significant down-regulation of *flaB1* and *flaB2* shown herein suggests that they are not suitable as control genes, especially for gene expression under the *in vivo* conditions.

### LPS and O-antigen synthesis

Unlike other none-lipopolysaccharide (LPS) spirochetes, *Leptospira* contains an intact and conserved biosynthesis system of LPS. Leptospiral LPS can activate host cells through TLR-2, which is distinct from other bacterial LPS [1], [46]. The structure of leptospiral lipid A and oligosaccharide can be modified [47], [48], which may attribute to the complicated diversity of more than 200 serovars. About 80% of ORFs involved in the leptospiral LPS synthesis pathway are similar to those of *E. coli*, which may indicate the highly conserved evolution of this genome locus [3]. However, unlike other gram-negative bacteria in which the lipid A biosynthesis genes are in an operon, these genes are scattered across the genome in *L. interrogans*. The O antigen synthesis genes remains clustered in the *rfb* operon with 103 kb in size [7].

Overall, genes in this category are evolutionarily conserved and showed limited changes. These changes included moderate up-regulation of UDP-N-acetylglucosamine acyltransferase gene (*lpxD2*, LA4326), several O-antigen synthesis genes [glucose-1-phosphate thymidylyltransferase (*rfbA*, LA3802), dTDP-glucose 4,6-dehydratase (*rfbB3*, LA1606), CDP-glucose 4,6-dehydratase (*rfbG*, LA1632)], and two of the O-antigen assembly genes [UDP-glucose lipid carrier transferase (*rfbP2*, LA2509), polysaccharide biosynthesis protein (*rfbX*, LA1649)], and down-regulation of O-antigen polymerase gene (*rfc*, LA1648). Note that the rate-limiting enzyme gene, *lpxA* (LA3949) had no change in our infection models.

### Sphingomyelinases and hemolysins

Leptospiral sphingomyelinases and hemolysins are hypothesized to degrade the host cell membrane and acquire nutrition [3]. Some were verified to be haemolytic enzymes or pore-forming cytotoxin [49], [50]. Pathogenic *L. interrogans* and *L. borgpetersenii* has 5 and 3 sphingomyelinase genes respectively, while non-pathogenic *L. biflexa* has no such genes [6]. Recent data showed that the major secreted sphingomyelinase C precursor gene (*sph2*, LA1029) is host-inducible during infection [51], [52]. Our microarray data showed that the *sph2* was expressed at extraordinary high level even in spirochetes cultivated in the EMJH medium. The *sph2*, along with another sphingomyelinase C precursor gene, *sph1* (LA1027), were further up-regulated either in the RPMI 1640 medium controls or upon interaction with macrophage cells. Up-regulation of these genes were likely due to changes in osmolarity, since it was previously shown that that expression of Sph2 increases when *L. interrogans* was grown in a physiologically relevant osmotic concentration [52].

Regarding non-sphingomyelinase hemolysins, one of the putative hemolysin genes, which is present only in pathogenic *Leptospira*, *tlyC* (LA3937), was up-regulated upon interaction with macrophages. The hemolytic activity of TlyC remains controversial. One earlier study showed that TlyC had hemolytic activity [50]. A recent study from another group reported that TlyC had no hemolytic activity, but it was a surface protein that mediates interaction with host extracellular matrix (ECM) [53]. Nevertheless, up-regulation of *tlyC* upon interaction with host cells observed in this study supports the hypothesis that it may be expressed during infection and facilitates the infection of *L. interrogans*.

### Other pathogenesis-related genes

Several adhesion or invasion-related genes were annotated in the original *L. interrogans* Serovar Lai Strain Lai 56601 genome, including *mce*, *invA*, *atsE* and *mviN* [3]. The precise functions for most of these genes are largely unknown, with exception for *mviN*. Recent study shows that MviN (MuiJ) of *E. coli* is a peptidoglycan lipid II flippase [54]. In this study, *mviN*, as well as another putative virulence factor, *mce*, were up-regulated in *L. interrogans* up interaction with macrophages.

*L. interrogans* has several additional virulence-associated genes such as a collagenase gene (LA0872), a PAF acetylhydrolase gene (*pafAH*, LA2144) [55], a von Willebrand factor type A gene (*vwa*, LA0697), and a paraoxonase gene (*pon*, LA0399). The homologs of *pafAH*, *vwa* and *pon* are also present in saprophyte *Leptospira* [7]. These genes were largely unchanged in this model, with the except that *vwa* and *pon* were transiently up-regulated upon interaction with murine macrophages (J samples).

Iron acquisition is one of the essential survival strategies many pathogens possess for establishing infection in mammalian hosts [56]. TonB-dependent receptors are associated with iron acquisition [57] and may transport heme and hemoprotein [58]. Some of these outer membrane receptor genes (LA0572, LA2641 and LA3258) were up-regulated, while another receptor gene (LA3242) was down-regulated (**Table S2**) upon interaction with macrophages. The iron resources of EMJH and RPMI 1640 medium controls (with 10% heat-inactivated FCS) are somewhat different. The available iron in the EMJH medium is free Fe^2+^, whereas in the mammalian cell culture medium, iron source is Fe^3+^ in transferrin (TRF, siderophilin) provided by serum. This difference may also affect leptospiral iron transportation and uptake. For example, *hemO* (LB186) in Clade 6, the virulence gene encoding heme oxygenase for iron acquisition from hemoglobin [59], [60], was transiently up-regulated only in the RPMI 1640 medium controls. This regulation could be due to a difference in iron status among the EMJH medium, the RPMI 1640 medium controls, and the macrophage-containing cell culture medium.

### Outer membrane proteins and lipoproteins

The major components on the surface of *Leptospira* are transmembrane OMPs and lipoproteins [39]. These OMPs are functionally and structurally important for nutrition uptake, signal transduction, cell stabilization, and immunogenicity [61]. A mutant lacking ompA-like outer membrane protein, Loa22 (LA0222), had been generated, and showed that this gene was essential for *L. interrogans* pathogenesis [62]. Several major OMPs had been employed for developing subunit vaccines or serological tests for leptospirosis [37], [63], [64], [65], [66], [67].

**Figure 6:**
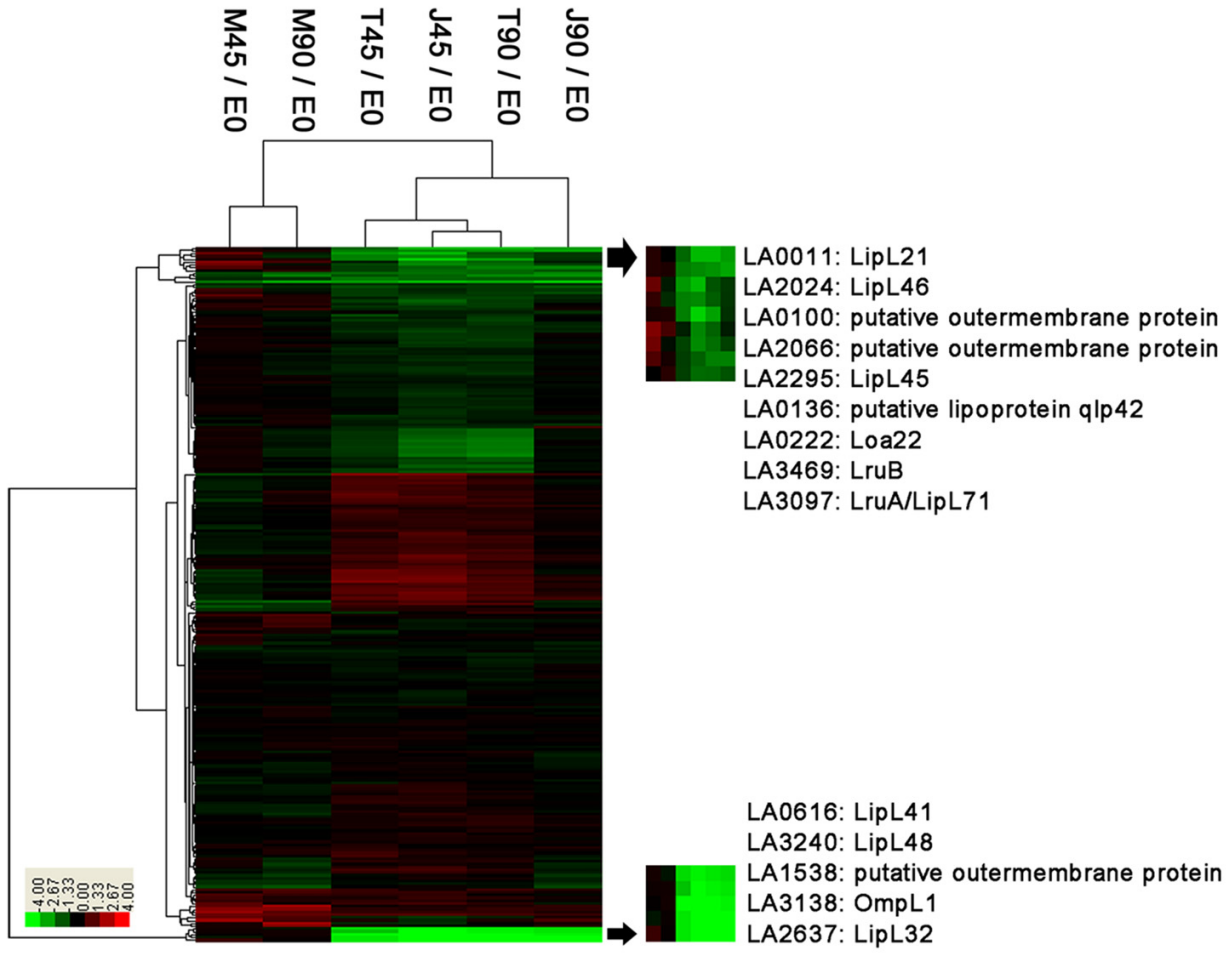
Sequential changes of the predicted leptospiral OMP genes. The balance between up-regulation (red) and down-regulation (green) indicated that *L. interrogans* altered its membrane in the infection models. The highly down-regulated transmembrane OMP and lipoprotein genes were clustered into two distinct subclades. The most highly down-regulated subclade included the genes of LipL41 (LA0616), LipL48 (LA3240), a putative OMPs (LA1538), OmpL1 (LA3138) and LipL32 (LA2637). Another highly down-regulated subclade included the genes of LipL21 (LA0011), LipL46 (LA2024), two putative outer membrane proteins (LA0100 and LA2066), LipL45 (LA2295), putative lipoprotein qlp42 (LA0136), Loa22 (LA0222), LruB (LA3469) and LruA/LipL71 (LA3097).

Based on genome annotation and lipoprotein prediction, there are at least 150 predicted lipoprotein genes (including predicted lipoproteins on inner and outer membrane) and about 100 predicted transmembrane OMP genes defined in the genomes of *L. interrogans* [3], [4], [11], [68], [69]. Cluster analysis of these 255 OMPs and lipoprotein genes revealed that half of these genes were up-regulated, while 40% of them were down-regulated (**Figure 6**). It was interesting to note that most of these changes were due to the interaction with macrophage rather than in the RPMI 1640 medium controls. Thus, it was likely that the membrane profiles underwent a series of dramatic changes upon interaction with macrophages. Most of the up-regulated genes were moderately regulated and almost all of these genes were putative transmembrane OMPs and putative lipoproteins not previously verified by proteomics [68]. One of the well-studied up-regulated genes was *lig* genes. The leptospiral *lig* genes encode several surface Lk90-like proteins containing immunoglobulin-like repeats, including LigA, LigB and LigC [7], [70], [71]. The *L. interrogans* Serovar Lai Strain Lai 56601 has LigB (LA3778) and LigC (LA3075) [3], whereas *L. interrogans* serovar Copenhageni strain Fiocruz L1-130 has LigA and LigB. Expression of *ligB* is up-regulated during infection, and LigB has been suggested as a putative virulence factor [72]. However, recent inactivation of *ligB* in *L. interrogans* Serovar Copenhageni Strain Fiocruz L1-130 did not affect leptospiral pathogenicity [9]. It was proposed that loss of LigB was compensated by the presence of LigA [8]. In this study, both expression of *ligB* and *ligC* in *L. interrogans* Serovar Lai Strain Lai 56601 were up-regulated either upon interaction with macrophages or in the RPMI 1640 medium controls alone. This result was consistent with the increased expression of *ligB* by evaluated temperature, host serum and during infection [15], [73]. Concurrent up-regulation of LigB and LigC also supported the functional compensatory hypothesis among LigA, LigB, and LigC.

Based on our microarray data, the most abundantly expressed genes in E0 samples were well-studied major transmembrane OMPs and surface lipoproteins (**Table, S2**), which was consistent with results of previous proteomics study [29]. The most interesting result was the dramatic down-regulation (10–50 fold) of these major OMPs upon interaction with macrophages (**Table 2**). These genes clustered into two unique subclades based on Euclidean distance (**Figure 6**). The most highly down-regulated subclade included the genes of LipL41 (LA0616), LipL48 (LA3240), a putative OMPs (LA1538), OmpL1 (LA3138) and LipL32 (LA2637). Another highly down-regulated subclade included the genes of LipL21 (LA0011), LipL46 (LA2024), two putative outer membrane proteins (LA0100 and LA2066), LipL45 (LA2295), putative lipoprotein qlp42 (LA0136), Loa22 (LA0222), LruB (LA3469) and LruA/LipL71 (LA3097). In addition, most of these genes were members of the above-mentioned Clade 1 in Cluster analysis of whole transcriptomics data (**Figure 2B**). The virulence gene *loa22* gene was down-regulated by 2–4 fold upon interaction with macrophages, and was included in the moderately down-regulated Clade 6. This down-regulation was contrary to an earlier report that expression of *loa22* was up-regulated by host serum [15]. Notably, this was not due to incubation in the RPMI 1640 medium controls, as down-regulation did not occur in M samples. The persistent down-regulation of these major OMPs was further validated by immunoblotting in this study (**Figure 5**).

**Table 2:** Category of leptospiral ORFs which were down-regulated at least 5-fold in infection models.

Clade ID	ORFID	M45/E0mean fold	J45/E0mean fold	T45/E0mean fold	M90/E0mean fold	J90/E0mean fold	T90/E0mean fold	Function and description of gene product
5	LA0011	1.5	−7.69	−3.13	1.01	−5.56	−7.69	putative lipoprotein (LipL21)
5	LA0100	3.17	−7.69	−5.26	0.75	0.54	−2.5	putative outermembrane protein
5	LA0242	0.72	−5.88	−6.25	1.17	−11.11	−5.88	cytochrome caa3 oxidase subunit II
5	LA0243	0.78	−5.26	−6.67	1.21	−10	−6.25	cytochrome C oxidase polypeptide I
5	LA0244	0.83	−3.45	−3.13	1.21	−6.25	−4.35	cytochrome C oxidase polypeptide III (Cytochrome AA3 subunit 3)
2	LA0296	−2.56	−4.17	−3.85	−5.88	−5.56	−4.17	alcohol dehydrogenase
5	LA0411	0.93	−5.88	−5	1.2	−9.09	−6.67	electron transfer flavoprotein alpha-subunit (Alpha-ETF; Electron transfer flavoprotein large subunit; ETFLS)
1	LA0412	0.84	−14.29	−11.11	1.19	−10	−14.29	electron transfer flavoprotein beta-subunit (Beta-ETF; Electron transfer flavoprotein small subunit; ETFSS)
1	LA0505	1.92	−9.09	−8.33	1.17	−8.33	−11.11	probable glycosyl hydrolase (OmpL47)
2	LA0587	0.86	−5	−4.76	−7.69	−9.09	−5.56	lactonizing lipase (Triacylglycerol lipase)
1	LA0616	1.26	−14.29	−11.11	0.82	−11.11	−12.5	outer membrane lipoprotein lipL41
5	LA0737	1.23	−11.11	−6.25	1.21	−4.17	−6.25	elongation factor Tu
1	LA0738	1.24	−14.29	−9.09	1.25	−7.14	−9.09	ribosomal protein S10
5	LA0739	1.18	−7.14	−5.88	1.36	−5.56	−5.56	ribosomal protein L3
5	LA0751	1.38	−3.33	−3.23	1.35	−5.56	−3.13	ribosomal protein L5
	LA0755	1.07	−2.56	−2.44	0.54	−5.56	−2.94	ribosomal protein L18
	LA0756	1.05	−2.7	−2.22	0.68	−5.26	−3.03	ribosomal protein S5
5	LA0757	0.88	−2.63	−3.85	0.78	−5.26	−4	ribosomal protein L30
	LA1084	1.21	−3.7	−2.04	0.66	0.89	−5	hypothetical protein
2	LA1101	−2.33	−4.35	−3.23	−5	−5.56	−4.55	succinyl-CoA synthetase alpha subunit
2	LA1102	0.54	−5	−4.35	−4.17	−5.56	−5	succinyl-CoA synthetase beta chain
5	LA1202	0.93	−3.85	−3.85	0.58	−5.26	−5	hypothetical protein
5	LA1313	0.91	−5.88	−5.56	0.83	−4.17	−4	glutamine synthetase (Glutamate–ammonia ligase)
1	LA1402	2.41	−6.25	−2.86	1.04	−16.67	−10	hypothetical protein
5	LA1403	−2.33	−3.7	−6.67	1.26	−3.85	−3.85	hypothetical protein
2	LA1471	−2.7	−3.33	−2.86	−8.33	−5.88	−3.7	pyrophosphate-energized vacuolar membrane proton pump (Pyrophosphate-energized inorganic pyrophosphatase; H+-PPase)
5	LA1532	0.52	−5	−3.45	0.8	−4	−3.33	fructose-bisphosphate aldolase (catalyzes the formation of glycerone phosphate and D-glyceraldehyde 3-phosphate from D-fructose 1,6-bisphosphate)
1	LA1538	1.05	−33.33	−20	1.22	−14.29	−20	putative outermembrane protein
1	LA1539	0.95	−50	−50	1.19	−12.5	−25	orotate phosphoribosyltransferase
4	LA1563	4.47	−6.25	−3.33	2.91	0.89	0.79	class II heat shock protein (HSP20)
4	LA1564	2.02	−11.11	−2.94	1.71	1.29	0.62	class I heat shock protein (HSP20)
5	LA1676	1.23	−4.76	−5.56	0.52	−2.94	−3.85	single-stranded DNA-binding protein
5	LA1677	1.15	−5	−5.26	1.05	−3.7	−4.35	ribosomal protein S18
	LA1678	1.1	−4.17	−5	2.44	0.52	−3.13	ribosomal protein L9
	LA1718	1.23	−4	0.55	0.65	0.88	−5	hypothetical protein
	LA1719	2.69	−6.67	−5	1.91	−2.08	−4.17	cysteine synthase (O-acetylserine sulfhydrylase; O-acetylserine (Thiol)-lyase)
2	LA1883	0.52	−2.94	−2.38	−4.17	−5	−4.55	hypothetical protein
2	LA1897	0.76	−6.25	−4.55	−3.33	−6.25	−5.26	succinate dehydrogenase (Converts succinate to fumarate as part of the TCA cycle. It is the only membrane bound enzyme in the TCA cycle)
	LA1901	1.25	−3.85	0.53	0.67	0.86	−5	hypothetical protein
2	LA1920	0.45	−6.25	−5.26	−5	−6.25	−5.56	RNA-binding protein
	LA2017	1.47	−16.67	−4.17	0.95	0.97	−5.56	periplasmic flagellin (flaB1)
4	LA2019	1.88	−20	−3.03	2.27	1.66	−3.03	periplasmic flagellin (flaB2)
5	LA2024	1.5	−4.55	−3.85	1.36	−6.25	−5	hypothetical protein (LipL46)
	LA2138	2.22	−6.25	−3.03	2.67	0.57	−3.57	hypothetical protein
5	LA2179	2.66	−5.56	−3.45	−2.5	−4.55	−5.26	recombinase A (catalyzes the hydrolysis of ATP in the presence of single-stranded DNA, the ATP-dependent uptake of single-stranded DNA by duplex DNA, and the ATP-dependent hybridization of homologous single-stranded DNAs)
	LA2181	1.27	−3.7	0.53	0.67	0.76	−5.26	hypothetical protein
	LA2239	1.25	−4	0.57	0.66	0.85	−5	hypothetical protein
	LA2295	1.35	−10	−4	1.41	0.59	−5.88	LipL45 protein
2	LA2309	−2.17	−10	−7.14	−9.09	−8.33	−6.25	long-chain-fatty-acid CoA ligase
	LA2360	0.89	−4.35	−4	−7.69	−9.09	−4.76	ribonucleotide-diphosphate reductase alpha subunit (Catalyzes the rate-limiting step in dNTP synthesis)
1	LA2418	0.98	−16.67	−9.09	−2.27	−4.76	−6.67	possible hook-associated protein, flagellin family
5	LA2458	0.55	−6.67	−5	1.08	−4.76	−5.26	hypothetical protein
1	LA2637	1.86	−33.33	−12.5	1.09	−20	−33.33	LipL32 protein
4	LA2654	4.01	−6.67	−4.55	3.22	3.47	1.05	10 kDa chaperonin (Protein CPN10) (Protein GROES) (Heat shock 10 kDa protein)
4	LA2655	2.17	−6.67	−2.86	1.8	1.56	0.64	60 kDa chaperonin (Protein Cpn60) (groEL protein) (Heat shock 58 kDa protein)
5	LA2781	1.06	−6.67	−4.35	0.84	−6.67	−6.67	ATP synthase F0, B subunit
2	LA2834	−3.57	−5.88	−5.56	−2.86	−5.56	−4.76	adenylate cyclase
2	LA2835	−3.45	−9.09	−6.67	−3.13	−6.25	−6.25	hypothetical protein (FMN-binding split barrel, related; Pyridoxamine 5′-phosphate oxidase-related, FMN-binding core)
2	LA2859	−2.27	−5	−3.23	−2.17	−2.86	−3.33	hypothetical protein
	LA2888	1.2	−4	0.56	0.71	0.9	−5	hypothetical protein
	LA3081	1.23	−3.85	0.54	0.67	0.87	−5	hypothetical protein
1	LA3138	0.8	−20	−12.5	1.12	−20	−16.67	transmembrane outer membrane protein L1 (OmpL1)
5	LA3143	0.97	−6.67	−3.85	0.87	−2.78	−4	acyl-CoA dehydrogenase
1	LA3240	1.19	−16.67	−8.33	1.2	−12.5	−14.29	hypothetical protein (LipL48)
2	LA3263	0.87	−4.76	−4.35	0.94	−8.33	−4.76	hypothetical protein
2	LA3264	1.28	−7.69	−5.56	1.16	−9.09	−6.67	hypothetical protein (Cytochrome c, monohaem; Cytochrome c, alcohol dehydrogenase-like subunit)
1	LA3265	0.72	−9.09	−10	0.86	−10	−9.09	hypothetical protein
2	LA3266	0.95	−8.33	−6.25	1.13	−12.5	−8.33	molybdopterin oxidoreductase
2	LA3267	1.35	−8.33	−6.67	1.19	−12.5	−8.33	molybdopterin oxidoreductase, iron-sulfur binding subunit
2	LA3268	0.81	−6.67	−5.56	1.12	−7.69	−7.69	cytochrome c3 (Cytochrome c7; Cytochrome c551.5)
5	LA3298	1.46	−7.14	−2.86	0.66	−3.13	−4.76	30S ribosomal protein S2 (Essential for binding of S1 to the small ribosomal subunit)
5	LA3379	2.26	−6.67	−3.45	0.7	−3.57	−4.35	flagellar filament outer layer protein A
	LA3380	2.45	−6.67	−2.7	2.57	0.8	−3.03	flagellar filament outer layer protein A
5	LA3417	0.75	−4.55	−4.17	0.54	−3.03	−5	30S ribosomal protein S12 (Important for translational accuracy. Interacts with and stabilizes bases of the 16S rRNA that are involved in tRNA selection in the A site and with the mRNA backbone. Located at the interface of the 30S and 50S subunits, it traverses the body of the 30S s)
5	LA3419	0.97	−4	−3.33	1.31	−5.26	−4.35	DNA-directed RNA polymerase beta' subunit (DNA-dependent RNA polymerase catalyzes the transcription of DNA into RNA using the four ribonucleoside triphosphates as substrates)
	LA3426	1.66	−5.26	−2.33	1.98	−2.22	−2.94	hypothetical protein (SecE subunit of protein translocation complex; Protein secE/sec61-gamma protein)
4	LA3705	2.88	−6.25	−3.23	1.39	1.2	0.86	chaperone protein dnaK
5	LA3707	1.7	−4.17	−5	1.02	0.65	−2.27	hypothetical protein
	LA3793	2.76	−6.67	−3.85	4.32	−2.08	−3.7	hypothetical protein (Acyl-CoA N-acyltransferase)
	LA3829	3.68	−8.33	−5.56	2.79	−3.33	−10	hypothetical protein
	LA3961	2.06	−6.25	−4.35	1.64	−2.04	−4.55	hypothetical protein (OmpL36)
5	LA4067	1.08	−5.26	−4.55	1.31	−4.55	−4.55	isocitrate dehydrogenase (Converts isocitrate to alpha ketoglutarate)
	LA4303	1.24	−3.85	0.54	0.69	0.95	−5	hypothetical protein
4	LB099	1.52	−14.29	−6.67	3.54	1.34	−2.27	hypothetical protein
5	LB106	2.55	−5.26	−4.17	−2.17	−2.27	−3.57	S-adenosyl-L-homocysteine hydrolase (catalyzes the formation of L-homocysteine from S-adenosyl-L-homocysteine)
2	LB327	0.73	−4.76	−3.85	−4	−7.14	−4.55	aconitate hydratase

The ORFs down-regulated at least 5-fold in J or T samples were included in this table. The mean fold values were inverted into negative reciprocal values when the fold changes were 0.5 or less. The supplementary annotations generated in this study were showed in brackets. Clade ID: the clade ID for the significantly regulated ORF defined in genome-wide cluster analysis (Figure 2B).

It is well established that leptospiral major OMPs are differentially expressed in semi-*in vivo* conditions or during infection [17], [18], [74]. We found that the expression levels of leptospiral OMPs were different in EMJH, Korthof and other leptospiral culture mediums (Date not shown). However, factors that contribute to differential regulation of these major surface proteins were largely unknown. Previous studies indicated that the common factors, including temperature, osmolarity, iron, or host serum, did not significantly influence expression of these major OMPs [12], [13], [14], [15], [59]. Our results indicated that the interaction with host cells was an important factor in triggering differential expression of major OMPs in *L. interrogans*, a phenomenon not achieviable using leptospiral culture media. Since most of these surface proteins are major antigens and the macrophage is an important antigen presenting cell of the host, down-regulation of this group of proteins was likely an immune evasion mechanism of *L. interrogans* [75].

### Identification of potential transcription factors involving in differential gene expression

The above-mentioned significant changes of gene expression, especially the dramatic down-regulations of the major OMPs, created a problem when determining which regulation systems were involved in differential gene expression. The first step to solve this problem is to identify the TFs which were directly involved in the major changes. More than forty sigma factors, anti-sigma factors, and anti-sigma factor antagonists were defined in the genome of *L. interrogans* Serovar Lai Strain Lai 56601. Their expression levels were unchangeable upon interaction with host cells in our microarray study (**Table S2**). Especially, Sigma S (RpoS), the sigma factor that plays a key role in differential gene expression in another well-studied spirochete, *B. burgdorferi*, is not present in the six released *Leptospira* genomes [76]. The anti-sigma factors can control sigma factors activity at post-translation level, and may be involved in the major OMPs regulation.

*B. burgdorferi* has only a few of specific TFs, while there are lots of specific TF homologs defined in the leptospiral genomes, which increased the possibility that specific TFs also played important roles in the regulation of the major OMPs. The leptospiral signal transduction proteins had recently been classified by domain-based rules in MiST2 database (http://mistdb.com/) [77]. In this database, the putative TFs were classified into several catalogs, such as one-component proteins, two-component proteins, and response regulators, *etc*, but not systematized into specific TF families named after their original function. In this study, the DBD definitions were obtained from InterPro integrative protein signature database by InterProScan program and well annotated by InterPro2Go. The InterPro database integrates PROSITE, PRINTS, Pfam, ProDom, SMART, TIGRFAMs, PIR superfamily, SUPERFAMILY Gene3D and PANTHER databases, which guaranteed the accuracy of the definition of the functional DBD domains [31]. All putative TFs were classified into specific TF families based on the original definition of the DBD domains, which enabled us to predict the potential function of the putative TFs. In addition, the phylogenic tree for each of the TF families was constructed based on the whole TF sequences, which helped us to compare the TF homologs within specific families, and identify the specific TFs that only existed in pathogenic *Leptospira*, which may be associated with leptospiral pathogenisis (**Figure 1B**).

**Figure 7:**
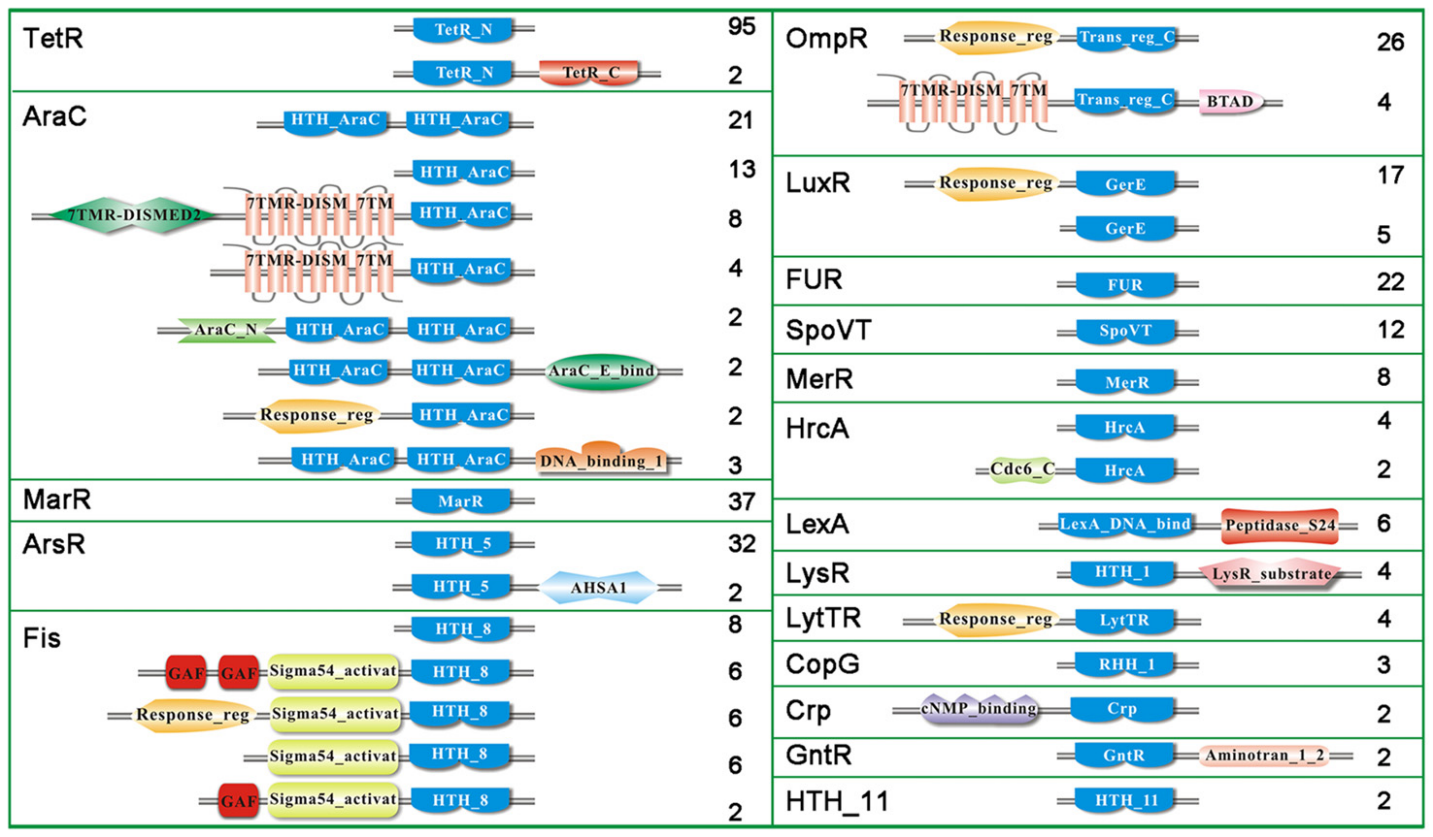
Domain structures of all predicted leptospiral specific transcription factors. Based on protein domain similarity, all specific TFs from the six released *Leptospira* genomes were classified into 18 TF families. The total number of TFs in each family was shown behind the structure model. The detailed TF catalog and evolutionary analysis can be inquired in Table S3.

Overall, the total number of specific TFs of non-pathogenic *L. biflexa* was almost twice than that of pathogenic *Leptospira* species (**Table S3**). That is, *L. biflexa* had about 100 specific TFs, while *L. interrogans* and *L. borgpetersenii* had less than 50 specific TFs. In addition, *L. biflexa* had higher proportions of TFs (TFs/ORFs) than pathogenic *Leptospira*, which is consistent with its strong survivability and high growth rate [6]. Based on domain analysis, 18 specific TF families were defined in six released leptospiral genomes (**Figure 7**). Several TF families were not found in all leptospiral genomes. The HTH_11 family existed only in *L. interrogans*, and the MerR, LytTR, LysR, Crp and GntR families existed only in *L. biflexa*. The CopG families existed in *L. borgpetersenii* and *L. biflexa*, but was absent in *L. interrogans*. (The previous definitions of CopG TFs in the genome of *L. interrogans* Lai 56601 were not precise, and there were no CopG TFs in the genome of *L. interrogans* Serovar Copenhageni Strain Fiocruz L1-130.) Based on the microarray date in this study, several specific TF genes with high expression levels were identified, such as LB333 of the OmpR family, LA3094 of the Fur family, LA1447 of the LexA family, LA0900 of the MarR family and LA3531 of the ArsR family, *etc*, which may contribute to the major regulation in our microarray study.

Most specific TF families of non-pathogenic *L. biflexa* were larger than those of the pathogenic *Leptospira*. The only exception was that the OmpR TF family of pathogenic *Leptospira* species (*L. interrogans and L. borgpetersenii*), which was larger than that of *L. biflexa*. Considering that the OmpR TF was first defined as a regulator of outer membrane porin genes (*ompC* and *ompF*) in *E. coli* [78], [79], it is possible that the leptospiral OmpR TFs were also involved in the regulation of the porins or other membrane proteins. Pathogenic *Leptospira* may regulate OMPs more efficiently than non-pathogenic *L. biflexa*. Furthermore, this OMP regulation may be related with leptospiral pathogenisis. If so, it would be consistent with the down-regulation of the major OMPs observed herein and previously [18].

**Figure 8:**
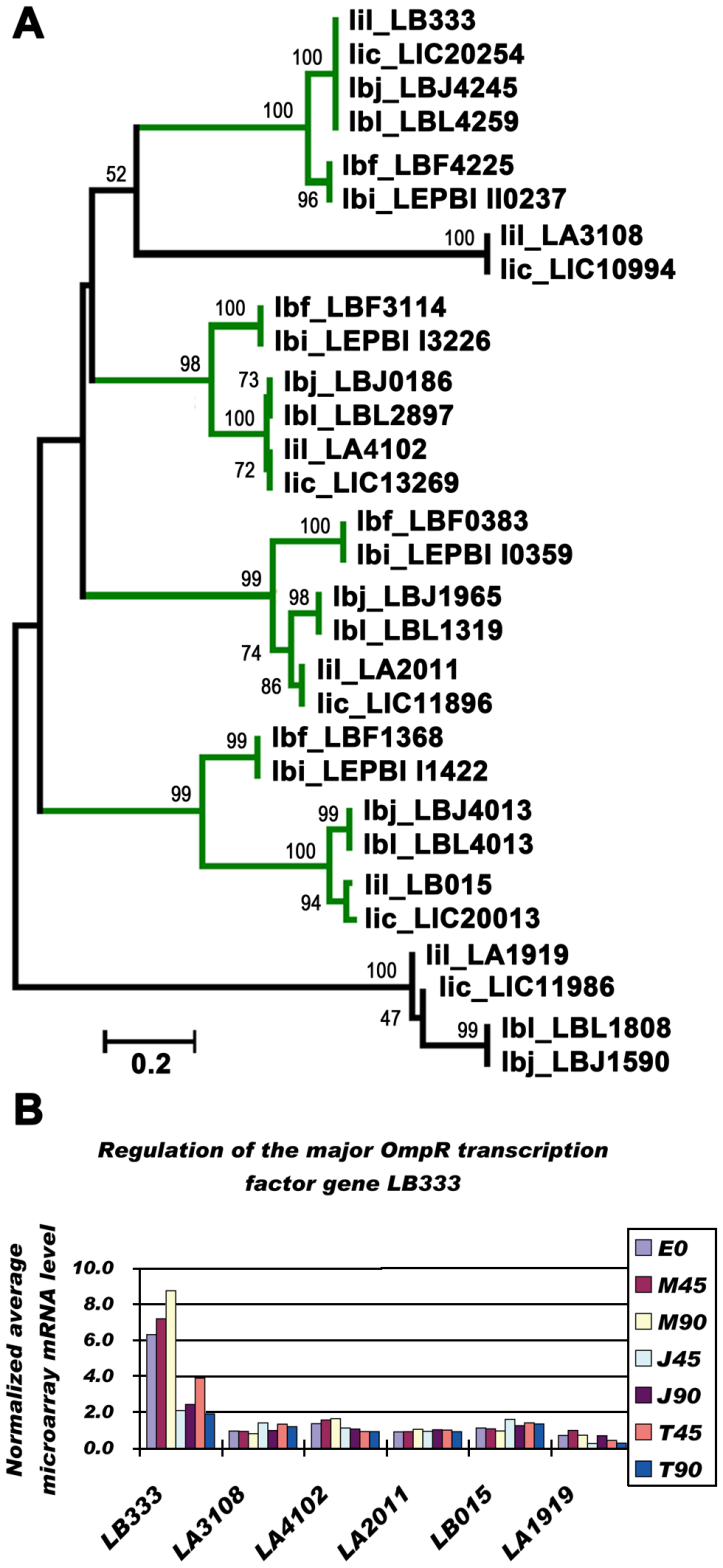
Molecular evolution and gene regulation of leptospiral OmpR transcription factors. The molecular evolutionary tree was constructed using the Neighbor-Joining method implemented in the MEGA 4.0 program with confidences of topology summarized from 1000 bootstrap replications based on the whole sequences of OmpRs. Only the bootstrap values larger than 50% were shown on the branches. Orthologous OmpRs sharing among all of the six *Leptospira* genomes syntenies were marked in a yellow green. lil: *L. interrogans* Serovar Lai Strain Lai 56601; lic: *L. interrogans* Serovar Copenhageni Strain Fiocruz L1-130; lbj: *L. borgpetersenii* Serovar Hardjo-bovis Strain JB197; lbl: *L. borgpetersenii* Serovar Hardjo-bovis Strain L550; lbf: *L. biflexa* Serovar Patoc Strain Patoc I (Ames); lbi: *L. biflexa* Serovar Patoc Strain Patoc I (Paris) (**A**). Gene regulation analysis of the OmpR TFs showed that LB333 was the unique OmpR TF gene which was highly-expressed in EMJH and RPMI 1640 medium (E0, M45 and M90), but significantly down-regulated in infection models (J45, J90, T45 and T90) (**B**).

The molecular phylogeny of OmpR family revealed four monophyletic origins in all six *Leptospira* spp. (**Figure 8A**). Two exceptions were that LA3108 homologs were only found in *L. interrogans*, and LA1919 homologs only existed in pathogenic *Leptospira* species (*L. interrogans and L. borgpetersenii*). Based on domain analysis (**Figure 7**), LA1919 was supposed to encode a TF with seven putative transmembrane regions, which was seldom present in prokaryotes but common in eukaryotes. Of note, LB333 was the most abundantly expressed TF gene and the only OmpR TF gene highly expressed in EMJH medium, but was down-regulated significantly upon interaction with macrophages (**Figure 8B**). Furthermore, it was concomitantly down-regulated with the group of major OMP genes including *ompL1*, *lipL32*, *lipL41*, *lipL48* and *ompL47*, implying LB333 contributed to the differential regulation of this group of genes.

### Concluding remarks

The global trancriptomic analyses of pathogens using a bacteria-host cell interaction model provide new understandings of immune evasion and pathogenesis for many bacterial pathogens [80], [81], [82]. Of note, a host-adapted model using a dialysis membrane chamber (DMC) for the Lyme disease spirochete, *B. burgdorferi*, has been used for genome-wide analysis of transcriptome in response to host-specific signals. However, this method can not be used for *L. interrogans* Serovar Lai Strain Lai 56601 as it can not survive and replicate within DMC (Date not shown). In this study, we adopted macrophage-derived cell models to analyze the leptospiral transcriptomic changes upon interaction with host cells or to host-specific signals. The adaptability to host microenvironments and immune evasion mechanism of *L. interrogans* revealed in this study were significantly different from those of the previous *in vitro* studies. Although the main virulence factors of *Leptospira* remain largely unknown due to the high difficulty of genetic manipulation, these significant changes of *L. interrogans* in different microenvironments led us to recommend that further research should be performed under conditions that imitate *in vivo* conditions as much as possible.

One of the major observations in this study was the dramatic down-regulation of major OMPs (e. g., LipL32, OmpL1, LipL41 and LipL48) upon interaction with host cells. This was in contrast to previous microarray analyses using various growth conditions, such as varied temperature, osmolarity, or the presence of host serum, which did not observe changes in expression of these genes. The fact that these genes, including LipL32, LipL41, and LipL21, *etc*, have been shown differentially regulated during infection, suggests that interaction with host is a key signal for regulating expression of these genes [17], [18]. Our observations in this study suggested that down-regulation of these major OMPs may be important for the immune evasion of pathogenic *Leptospira*, a strategy similar to *B. burgdorferi* [83], [84]. These changes in the OMPs profile also suggested that host cells induce a substantial change in surface protein profile, which is important when considerating vaccine candidates against leptospirosis [80].

Regulation of the major OMP genes of *L. interrogans* appeared to be different from that of *B. burgdorferi*. The expression levels of the major leptospiral OMPs, such as LipL32, LipL41, and OmpL1, *etc*, were relatively stable and not responsive to common environmental cues, whereas the major surface lipoprotein OspC of *B. burgdorferi*, is induced by elevated temperature, lowered pH, higher cell density, the presence of CO_2_ or host serum. Regulation of *ospC* is directly controlled by sigma S (RpoS), which is further controlled by transcription factors including Rrp2, RpoN (sigma N), and BosR [76], [85]. *L. interrogans* does not have *rpoS* homologue in its genome. The specific TFs may play important roles in gene regulation of the major leptospiral OMPs. Co-regulation of LB333 with the major OMPs suggests that LB333 may be involved in such regulation. In addition, the comparative and evolutionary relationship of all the leptospiral specific TFs revealed in this study also facilitates further research on identifying the regulation networks in *Leptospira* spp.

This report focused on the common regulation of *L. interrogans* Strain Lai 56601 infecting the murine and human macrophage cell lines, especially the persistent down-regulation of the major OMPs. However, several differences in the transcriptomic changes of *L. interrogans* Strain Lai 56601 infecting the murine vs. human macrophage cell lines were also observed. Cluster analysis on the global transcriptomics (Figure 2A) showed that the J90/E0 was somewhat similar to M45/E0 and M90/E0, which indicated that most of the gene regulations in *L. interrogans* upon interaction with murine macrophages (J90) were not persistent changes. For example, several genes of anti-anti-sigma factor (anti-sigma factor antagonists), ArsR TF, heat shock proteins, and chaperonins in Clade 4 were differently regulated in J and T samples, which may contribute to the differential regulations upon interaction with murine vs. human macrophages both at transcription level and at translation level. In addition, the major flagellin genes, *flaB1/2* (LA2017 and LA2019), were persistently down-regulated in T samples, but only transiently down-regulated in J samples. Whether this difference contributed to the different behaviors of *L. interrogans* infecting murine vs. human macrophages remains unclear [23].

One limitation of our study was that macrophage cell lines instead of primary macrophages were used as infection models. However, our previous study had revealed that the behaviors of *L. interrogans* Strain Lai 56601 in the immortalized macrophage models were same as those in the primary macrophage models [23]. In addition, the high homogeneity and culturability of cell lines guaranteed the data reliability and repeatability in the highly sensitive microarray analysis. Therefore, only macrophage cell lines were employed as infection models in this leptospiral transcriptomics research. Another limitation was that the transcriptional responses revealed by microarray, qRT-PCR and Western blotting in this study only displayed the average and general regulations of all leptospiral cells infecting host cells. In fact, there were some individual differences among the invasive leptospiral cells morphologically [23]. Further cellular proteome analysis may complement the understanding of the individual regulation of pathogenic *Leptospia* during infection [86].

## Supporting Information

Table S1 Primers for quantitative real-time RT-PCR validation.
(0.03 MB DOC)

Table S2 The microarray data summary of the *L. interrogans* Serovar Lai Strain Lai 56601.
(0.48 MB ZIP)

Table S3 Catalog and evolutionary analysis of all leptospiral specific transcription factors.
(0.08 MB ZIP)

## References

[1] Bharti AR, Nally JE, Ricaldi JN, Matthias MA, Diaz MM (2003). Leptospirosis: a zoonotic disease of global importance.. Lancet Infect Dis.

[2] Levett PN (2001). Leptospirosis.. Clin Microbiol Rev.

[3] Ren SX, Fu G, Jiang XG, Zeng R, Miao YG (2003). Unique physiological and pathogenic features of *Leptospira interrogans* revealed by whole-genome sequencing.. Nature.

[4] Nascimento AL, Ko AI, Martins EA, Monteiro-Vitorello CB, Ho PL (2004). Comparative genomics of two *Leptospira interrogans* serovars reveals novel insights into physiology and pathogenesis.. J Bacteriol.

[5] Bulach DM, Zuerner RL, Wilson P, Seemann T, McGrath A (2006). Genome reduction in *Leptospira borgpetersenii* reflects limited transmission potential.. Proc Natl Acad Sci USA.

[6] Picardeau M, Bulach DM, Bouchier C, Zuerner RL, Zidane N (2008). Genome sequence of the saprophyte *Leptospira biflexa* provides insights into the evolution of *Leptospira* and the pathogenesis of leptospirosis.. PLoS ONE.

[7] Xue F, Yan J, Picardeau M (2009). Evolution and pathogenesis of *Leptospira* spp.: lessons learned from the genomes.. Microbes Infect.

[8] Ko A, Goarant C, Picardeau M (2009). *Leptospira*: the dawn of the molecular genetics era for an emerging zoonotic pathogen.. Nat Rev Microbiol.

[9] Croda J, Figueira CP, Wunder EA, Santos CS, Reis MG (2008). Targeted mutagenesis in pathogenic *Leptospira* species: disruption of the LigB gene does not affect virulence in animal models of leptospirosis.. Infect Immun.

[10] Murray GL, Srikram A, Hoke DE, Wunder EA, Henry R (2009). Major surface protein LipL32 is not required for either acute or chronic infection with *Leptospira interrogans*.. Infect Immun.

[11] Nascimento AL, Verjovski-Almeida S, Van Sluys MA, Monteiro-Vitorello CB, Camargo LE (2004). Genome features of *Leptospira interrogans* serovar Copenhageni.. Braz J Med Biol Res.

[12] Lo M, Bulach DM, Powell DR, Haake DA, Matsunaga J (2006). Effects of temperature on gene expression patterns in *Leptospira interrogans* serovar Lai as assessed by whole-genome microarrays.. Infect Immun.

[13] Qin JH, Sheng YY, Zhang ZM, Shi YZ, He P (2006). Genome-wide transcriptional analysis of temperature shift in *L. interrogans* serovar lai strain 56601.. BMC Microbiol.

[14] Matsunaga J, Lo M, Bulach DM, Zuerner RL, Adler B (2007). Response of *Leptospira interrogans* to physiologic osmolarity: relevance in signaling the environment-to-host transition.. Infect Immun.

[15] Patarakul K, Lo M, Adler B (2010). Global transcriptomic response of *Leptospira interrogans* serovar Copenhageni upon exposure to serum.. BMC Microbiol.

[16] Choy HA, Kelley MM, Chen TL, Moller AK, Matsunaga J (2007). Physiological osmotic induction of *Leptospira interrogans* adhesion: LigA and LigB bind extracellular matrix proteins and fibrinogen.. Infect Immun.

[17] Nally JE, Whitelegge JP, Bassilian S, Blanco DR, Lovett MA (2007). Characterization of the outer membrane proteome of *Leptospira interrogans* expressed during acute lethal infection.. Infect Immun.

[18] Monahan AM, Callanan JJ, Nally JE (2008). Proteomic analysis of *Leptospira interrogans* shed in urine of chronically infected hosts.. Infect Immun.

[19] Cinco M, Banfi E, Soranzo MR (1981). Studies on the interaction between macrophages and leptospires.. J Gen Microbiol.

[20] Wang B, Sullivan JA, Sullivan GW, Mandell GL (1984). Role of specific antibody in interaction of leptospires with human monocytes and monocyte-derived macrophages.. Infect Immun.

[21] Merien F, Baranton G, Perolat P (1997). Invasion of Vero cells and induction of apoptosis in macrophages by pathogenic *Leptospira interrogans* are correlated with virulence.. Infect Immun.

[22] Jin D, Ojcius DM, Sun D, Dong H, Luo Y (2009). *Leptospira interrogans* induces apoptosis in macrophages via caspase-8- and caspase-3-dependent pathways.. Infect Immun.

[23] Li S, Ojcius DM, Liao S, Li L, Xue F (2010). Replication or death: distinct fates of pathogenic *Leptospira* strain Lai within macrophages of human or mouse origin.. Innate Immun.

[24] Davis JM, Haake DA, Ramakrishnan L (2009). *Leptospira interrogans* stably infects zebrafish embryos, altering phagocyte behavior and homing to specific tissues.. PLoS Negl Trop Dis.

[25] Ellis WA, Thiermann AB (1986). Isolation of *Leptospira interrogans* serovar bratislava from sows in Iowa.. Am J Vet Res.

[26] Zuerner RL (2005). Laboratory maintenance of pathogenic *Leptospira*.. Curr Protoc Microbiol Chapter.

[27] Bernstein J, Khodursky A, Lin P, Sue L, Cohen S (2002). Global analysis of mRNA decay and abundance in *Escherichia coli* at single-gene resolution using two-color fluorescent DNA microarrays.. Proc Natl Acad Sci USA.

[28] Li L, Ojcius DM, Yan J (2007). Comparison of invasion of fibroblasts and macrophages by high- and low-virulence *Leptospira* strains: colonization of the host-cell nucleus and induction of necrosis by the virulent strain.. Arch Microbiol.

[29] Cullen PA, Cordwell SJ, Bulach DM, Haake DA, Adler B (2002). Global analysis of outer membrane proteins from *Leptospira interrogans* serovar Lai.. Infect Immun.

[30] Eisen MB, Spellman PT, Brown PO, Botstein D (1998). Cluster analysis and display of genome-wide expression patterns.. Proc Natl Acad Sci USA.

[31] Quevillon E, Silventoinen V, Pillai S, Harte N, Mulder N (2005). InterProScan: protein domains identifier.. Nucleic Acids Res.

[32] Tamura K, Dudley J, Nei M, Kumar S (2007). MEGA4: Molecular Evolutionary Genetics Analysis (MEGA) software version 4.0.. Mol Biol Evol.

[33] Pinne M, Haake DA (2009). A comprehensive approach to identification of surface-exposed, outer membrane-spanning proteins of *Leptospira interrogans*.. PLoS ONE.

[34] Shang ES, Summers TA, Haake DA (1996). Molecular cloning and sequence analysis of the gene encoding LipL41, a surface-exposed lipoprotein of pathogenic *Leptospira* species.. Infect Immun.

[35] Haake DA, Matsunaga J (2002). Characterization of the leptospiral outer membrane and description of three novel leptospiral membrane proteins.. Infect Immun.

[36] Haake DA, Champion CI, Martinich C, Shang ES, Blanco DR (1993). Molecular cloning and sequence analysis of the gene encoding OmpL1, a transmembrane outer membrane protein of pathogenic *Leptospira* spp.. J Bacteriol.

[37] Haake D, Mazel M, McCoy A, Milward F, Chao G (1999). Leptospiral outer membrane proteins OmpL1 and LipL41 exhibit synergistic immunoprotection.. Infect Immun.

[38] Haake DA, Chao G, Zuerner RL, Barnett JK, Barnett D (2000). The leptospiral major outer membrane protein LipL32 is a lipoprotein expressed during mammalian infection.. Infect Immun.

[39] Cullen PA, Xu X, Matsunaga J, Sanchez Y, Ko AI (2005). Surfaceome of *Leptospira* spp.. Infect Immun.

[40] Boylan JA, Lawrence KA, Downey JS, Gherardini FC (2008). *Borrelia burgdorferi* membranes are the primary targets of reactive oxygen species.. Mol Microbiol.

[41] Hassett DJ, Cohen MS (1989). Bacterial adaptation to oxidative stress: implications for pathogenesis and interaction with phagocytic cells.. FASEB J.

[42] Loewen P (1996). Probing the structure of catalase HPII of *Escherichia coli*–a review.. Gene.

[43] Murgia R, Garcia R, Cinco M (2002). Leptospires are killed in vitro by both oxygen-dependent and -independent reactions.. Infect Immun.

[44] Lo M, Cordwell SJ, Bulach DM, Adler B (2009). Comparative transcriptional and translational analysis of leptospiral outer membrane protein expression in response to temperature.. PLoS Negl Trop Dis.

[45] Charon NW, Goldstein SF (2002). Genetics of motility and chemotaxis of a fascinating group of bacteria: The spirochetes.. Annual Review of Genetics.

[46] Werts C, Tapping RI, Mathison JC, Chuang TH, Kravchenko V (2001). Leptospiral lipopolysaccharide activates cells through a TLR2-dependent mechanism.. Nat Immunol.

[47] Que-Gewirth NL, Ribeiro AA, Kalb SR, Cotter RJ, Bulach DM (2004). A methylated phosphate group and four amide-linked acyl chains in *Lleptospira interrogans* lipid A. The membrane anchor of an unusual lipopolysaccharide that activates TLR2.. J Biol Chem.

[48] Nally JE, Chow E, Fishbein MC, Blanco DR, Lovett MA (2005). Changes in lipopolysaccharide O antigen distinguish acute versus chronic *Leptospira interrogans* infections.. Infect Immun.

[49] Lee SH, Kim S, Park SC, Kim MJ (2002). Cytotoxic activities of *Leptospira interrogans* hemolysin SphH as a pore-forming protein on mammalian cells.. Infect Immun.

[50] Zhang YX, Geng Y, Bi B, He JY, Wu CF (2005). Identification and classification of all potential hemolysin encoding genes and their products from *Leptospira interrogans* serogroup Icterohaemorrhagiae serovar Lai.. Acta Pharmacologica Sinica.

[51] Artiushin S, Timoney JF, Nally J, Verma A (2004). Host-inducible immunogenic sphingomyelinase-like protein, Lk73.5, of *Leptospira interrogans*.. Infect Immun.

[52] Matsunaga J, Medeiros MA, Sanchez Y, Werneid KF, Ko AI (2007). Osmotic regulation of expression of two extracellular matrix-binding proteins and a haemolysin of *Leptospira interrogans*: differential effects on LigA and Sph2 extracellular release.. Microbiology.

[53] Eneas C, Angela SB, Ricardo MG, Aurora MC, Pricila H (2009). Leptospiral TlyC is an extracellular matrix-binding protein and does not present hemolysin activity.. FEBS letters.

[54] Ruiz N (2008). Bioinformatics identification of MurJ (MviN) as the peptidoglycan lipid II flippase in *Escherichia coli*.. Proc Natl Acad Sci USA.

[55] Yang JW, Zhang YX, Xu J, Geng Y, Chen XY (2009). Serum activity of platelet-activating factor acetylhydrolase is a potential clinical marker for leptospirosis pulmonary hemorrhage.. PLoS ONE.

[56] Guegan R, Camadro JM, Saint Girons I, Picardeau M (2003). *Leptospira* spp. possess a complete haem biosynthetic pathway and are able to use exogenous haem sources.. Mol Microbiol.

[57] Louvel H, Bommezzadri S, Zidane N, Boursaux-Eude C, Creno S (2006). Comparative and functional genomic analyses of iron transport and regulation in *Leptospira* spp.. J Bacteriol.

[58] Asuthkar S, Velineni S, Stadlmann J, Altmann F, Sritharan M (2007). Expression and characterization of an iron-regulated hemin-binding protein, HbpA, from *Leptospira interrogans* serovar Lai.. Infect Immun.

[59] Murray GL, Ellis KM, Lo M, Adler B (2008). *Leptospira interrogans* requires a functional heme oxygenase to scavenge iron from hemoglobin.. Microbes Infect.

[60] Murray GL, Srikram A, Henry R, Puapairoj A, Sermswan RW (2009). *Leptospira interrogans* requires heme oxygenase for disease pathogenesis.. Microbes Infect.

[61] Cullen PA, Haake DA, Adler B (2004). Outer membrane proteins of pathogenic spirochetes.. FEMS Microbiol Rev.

[62] Ristow P, Bourhy P, McBride FWD, Figueira CP, Huerre M (2007). The OmpA-like protein Loa22 is essential for leptospiral virulence.. PLoS Pathog.

[63] Seixas FK, da Silva EF, Hartwig DD, Cerqueira GM, Amaral M (2007). Recombinant Mycobacterium bovis BCG expressing the LipL32 antigen of *Leptospira interrogans* protects hamsters from challenge.. Vaccine.

[64] Croda J, Ramos JGR, Matsunaga J, Queiroz A, Homma A (2007). *Leptospira* immunoglobulin-like proteins as a serodiagnostic marker for acute leptospirosis.. J Clin Microbiol.

[65] Dong H, Hu Y, Xue F, Sun D, Ojcius D (2008). Characterization of the ompL1 gene of pathogenic *Leptospira* species in China and cross-immunogenicity of the OmpL1 protein.. BMC Microbiol.

[66] Yan W, Faisal SM, P. MS, Divers TJ, Barr SC (2009). Immunogenicity and protective efficacy of recombinant *Leptospira* immunoglobulin-like protein B (rLigB) in a hamster challenge model.. Microbes Infect.

[67] Luo D, Xue F, Ojcius DM, Zhao J, Mao Y (2009). Protein typing of major outer membrane lipoproteins from Chinese pathogenic *Leptospira* spp. and characterization of their immunogenicity.. Vaccine.

[68] Setubal JC, Reis M, Matsunaga J, Haake DA (2006). Lipoprotein computational prediction in spirochaetal genomes.. Microbiology.

[69] Viratyosin W, Ingsriswang S, Pacharawongsakda E, Palittapongarnpim P (2008). Genome-wide subcellular localization of putative outer membrane and extracellular proteins in *Leptospira interrogans* serovar Lai genome using bioinformatics approaches.. BMC Genomics.

[70] Palaniappan RU, Chang YF, Jusuf SS, Artiushin S, Timoney JF (2002). Cloning and molecular characterization of an immunogenic LigA protein of *Leptospira interrogans*.. Infect Immun.

[71] Cerqueira GM, McBride AJA, Picardeau M, Ribeiro SG, Moreira AN (2009). Distribution of the Leptospiral immunoglobulin-like (Lig) genes in pathogenic *Leptospira* spp. and application of ligB to typing leptospiral isolates.. J Med Microbiol.

[72] Lin YP, Raman R, Sharma Y, Chang YF (2008). Calcium binds to leptospiral immunoglobulin-like protein, LigB, and modulates fibronectin binding.. J Biol Chem.

[73] Matsunaga J, Sanchez Y, Xu X, Haake DA (2005). Osmolarity, a key environmental signal controlling expression of leptospiral proteins LigA and LigB and the extracellular release of LigA.. Infect Immun.

[74] Nally JE, Timoney JF, Stevenson B (2001). Temperature-regulated protein synthesis by *Leptospira interrogans*.. Infect Immun.

[75] Blasi E, Ardizzoni A, Colombari B, Neglia R, Baschieri C (2007). NF-kB activation and p38 phosphorilation in microglial cells infected with *Leptospira* or exposed to partially purified leptospiral lipoproteins.. Microb Pathog.

[76] Hubner A, Yang X, Nolen DM, Popova TG, Cabello FC (2001). Expression of *Borrelia burgdorferi* OspC and DbpA is controlled by a RpoN-RpoS regulatory pathway.. Proc Natl Acad Sci USA.

[77] Ulrich LE, Zhulin IB (2010). The MiST2 database: a comprehensive genomics resource on microbial signal transduction.. Nucleic Acids Res.

[78] Mizuno T, Mizushima S (1990). Signal transduction and gene regulation through the phosphorylation of two regulatory components: the molecular basis for the osmotic regulation of the porin genes.. Mol Microbiol.

[79] Qin L, Yoshida T, Inouye M (2001). The critical role of DNA in the equilibrium between OmpR and phosphorylated OmpR mediated by EnvZ in *Escherichia coli*.. Proc Natl Acad Sci USA.

[80] Grifantini R, Bartolini E, Muzzi A, Draghi M, Frigimelica E (2002). Previously unrecognized vaccine candidates against group B meningococcus identified by DNA microarrays.. Nat Biotechnol.

[81] Livengood JA, Schmit VL, Gilmore RD (2008). Global transcriptome analysis of *Borrelia burgdorferi* during association with human neuroglial cells.. Infect Immun.

[82] Fontan P, Aris V, Ghanny S, Soteropoulos P, Smith I (2008). Global transcriptional profile of *Mycobacterium tuberculosis* during THP-1 human macrophage infection.. Infect Immun.

[83] Liang FT, Jacobs MB, Bowers LC, Philipp MT (2002). An immune evasion mechanism for spirochetal persistence in Lyme borreliosis.. J Exp Med.

[84] Crother TR, Champion CI, Wu XY, Blanco DR, Miller JN (2003). Antigenic composition of *Borrelia burgdorferi* during infection of SCID mice.. Infect Immun.

[85] Ouyang Z, Kumar M, Kariu T, Haq S, Goldberg M (2009). BosR (BB0647) governs virulence expression in *Borrelia burgdorferi*.. Mol Microbiol.

[86] Malmstrom J, Beck M, Schmidt A, Lange V, Deutsch EW (2009). Proteome-wide cellular protein concentrations of the human pathogen *Leptospira interrogans*.. Nature.

