# Supplementary material for: Transcriptional Responses of Leptospira interrogans to Host Innate Immunity: Significant Changes in Metabolism, Oxygen Tolerance, and Outer Membrane
Source: PLoS Negl Trop Dis. 2010 Oct 26;4(10):e857. doi: 10.1371/journal.pntd.0000857 (PMC2964297; doi:10.1371/journal.pntd.0000857)
Supplement: Table S1 — Primers for quantitative real-time RT-PCR validation. (0.03 MB DOC) [file pntd.0000857.s001.doc]

**Table S1. Primers for quantitative real-time RT-PCR verification**

| Gene | Forward primer sequence | Reverse primer sequence | Amplicon  Size (bp) | Annealing  Temp (℃) |
| --- | --- | --- | --- | --- |
| LA0031 | AACCGACGCAGATACAAAGAAAAA | ACCAGCCGCTAAGGAAAGTGAA | 101 | 57 |
| LA0237(*ctaB*) | CCTTTGATCGGTTACGCTGCTA | GCCGGGGTCCATAAGAAAATCATC | 88 | 58 |
| LA0737(*tuf*) | GACAACATCGGCGCTCTTCTTC | GACGTCCGCCTTCATCCTTAGTTA | 142 | 58 |
| LA3138(*ompL1*) | GGCCAGAGTAACCGATAAAGGACA | GATCGGATATGCTGGAAAAGGAGA | 130 | 57 |
| LA3189 | ACTTTCCGGTCCTTGGGTCCTTAC | GCCTGAGATCGTTTGAATGCTGAG | 122 | 60 |
| LB191 | AATATGTGAGCGGTGCGGTTTC | CTTGATAGGCGTTCCCAGTAGTCG | 98 | 58 |
